# Genomic characterization of a novel, widely distributed *Mycoplasma* species “*Candidatus* Mycoplasma mahonii” associated with the brittlestar *Gorgonocephalus chilensis*

**DOI:** 10.1371/journal.pone.0290305

**Published:** 2023-08-24

**Authors:** Oluchi Aroh, Mark R. Liles, Kenneth M. Halanych

**Affiliations:** 1 Department of Biological Sciences, Auburn University, Auburn, AL, United States of America; 2 Centre for Marine Science, University of North Carolina Wilmington, Wilmington, NC, United States of America; Yenepoya University, INDIA

## Abstract

Symbiotic relationships are ubiquitous throughout the world’s oceans, yet for many marine organisms, including those in the high latitudes, little is understood about symbiotic associations and functional relationships. From a recently determined genome sequence of a filter-feeding basket star from Argentina, *Gorgonocephalus chilensis*, we discovered a novel *Mycoplasma* species with a 796Kb genome (CheckM completeness of 97.9%, G+C content = 30.1%). Similar to other *Mycoplasma* spp. within Mycoplasmatota, genomic analysis of the novel organism revealed reduced metabolic pathways including incomplete biosynthetic pathways, suggesting an obligate association with their basket star host. Results of 16S rRNA and multi-locus phylogenetic analyses revealed that this organism belonged to a recently characterized non-free-living lineage of *Mycoplasma* spp. specifically associated with marine invertebrate animals. Thus, the name “*Candidatus* Mycoplasma mahonii” is proposed for this novel species. Based on 16S rRNA PCR-screening, we found that *Ca*. M. mahonii also occurs in *Gorgonocephalus eucnemis* from the Northwest Pacific and other *Gorgonocephalus chilensis* from Argentinian waters. The level of sequence conservation within *Ca*. M. mahonii is considerable between widely disparate high-latitude *Gorgonocephalus* species, suggesting that oceanic dispersal of this microbe may be greater than excepted.

## Introduction

*Mycoplasma* species are one of the smallest and simplest self-replicating organisms, with a very reduced genome size that can range from about 540 to 1300 Kb. Mycoplasmas possess the minimum set of genes essential for growth and replication and evolved from the *Bacillus/Clostridium* branch of Gram-positive eubacteria by reductive evolution [1, 2]. These bacteria lack a cell wall, a feature responsible for their pleomorphism and their resistance to some antibiotics [2].

Mycoplasmas encompass over 100 species and usually live in close association with their plant or animal host(s) to fulfill nutritional requirements [3]. Pathogenic mycoplasmas are responsible for numerous respiratory infections in humans such as pneumonia, as well as other conditions including pelvic inflammatory disease and urethritis [4]. In addition, the existence of mycoplasmas has been successfully documented in marine organisms including fishes (where they mainly colonize the intestines, gills, liver, and kidney) [2, 5, 6], cold-water corals [7], lobster [8], octopus [9, 10], abalone [11, 12] and squid [10]. They have also been found to be associated with the microbiota of echinoderms such as the blue bat star *Patiria Pectnifera* [13]. In these studies, mycoplasmas were in a commensal association with their host organism except in salmonids [2] where a mutualistic association was observed.

In this study, we report the discovery of a *Mycoplasma* with a 796kb genome (CheckM completeness of 97.9%) in the tissue of *Gorgonocephalus chilensis*, a filter-feeding basket star. To promote understanding of *Mycoplasma* spp. diversity, and symbiosis with marine invertebrates, we explored the new species’ genomic composition and inferred metabolic capabilities. Additionally, given the understudied environment from which this cold-water filter-feeding echinoderm was discovered, we explored the degree of relatedness between *Ca*. M. mahonii and other *Mycoplasma* spp. using 16S rRNA and multilocus phylogenetic analyses. and whether this novel species occurred in other basket stars. To the best of our knowledge, this is the first genetic isolation and characterization of mycoplasmas in the *Gorgonocephalus* genus and the name *Candidatus* Mycoplasma mahonii is proposed for this novel species.

## Materials and methods

### Genome sequencing

Genomic DNA of *Gorgonocephalus chilensis* (Ophiuroidea, Euryalida, Gorgonocephalidae) was extracted from an individual sampled in Argentinian waters during an *ARSV Laurence M*. *Gould* expedition (LMG-0605, May 2006, latitude; 54°49, longitude -60°16, depth 110m). Tissue was stored at -80°C and subsequently sent to the University of Arizona Genomics Institute where DNA was isolated and sequenced using PacBio CCS long read technology using the protocol outlined below.

High molecular weight (HMW) DNA was extracted from ground tissue in extraction buffer with Tris HCl buffer 0.1M pH 8.0, EDTA 0.1M pH8, SDS 1%, and Proteinase K in 50°C for 30 minutes. The mixture was spun down and the aqueous phase transferred to a new tube. Next, 5M Potassium acetate was added, precipitated on ice, and spun down. After centrifugation, the supernatant was gently extracted with 24:1 chloroform: isoamyl alcohol. The upper phase was transferred to a new tube and DNA precipitated with isopropanol. DNA was collected by centrifugation, washed with 70% ethanol, air dried, and dissolved thoroughly in 1x TE followed by RNAse treatment. The DNA purity was measured using Nanodrop, DNA concentration was measured with Qubit HS kit (Invitrogen), and DNA size was validated by the Femto Pulse System (Agilent). The extracted HMW DNA was sheared to appropriate size range (10–30 kb) using Megaruptor 3 (Diagenode). The sequencing library was constructed following manufacturers protocols using SMRTbell Express Template Prep kit 2.0. The final library was size selected on a Blue Pippin (Sage Science) using S1 marker with a 10–25 kb size selection. The recovered final library was quantified with Qubit HS kit (Invitrogen) and size checked on Femto Pulse System (Agilent). The sequencing library was prepared with PacBio Sequel II Sequencing kit 2.0 for HiFi library, loaded to 8M SMRT cells, and sequenced in CCS mode in the Sequel II instrument for 30 hours.

Raw reads were assembled using HiFiasm [14] (N50 = 1057833bp, Genome size = 3.5GB). The assembled genome of *G*. *chilensis* was screened for bacterial 16S rRNA by BLASTn against the NCBI’s 16S rRNA database [15]. Contigs that matched to 16S rRNA genes were assigned taxonomic labels using Kraken2 Silva database v.138 [16], with default parameters, and were further analyzed. For all software used herein, default parameters were employed unless otherwise noted.

Completeness of each matched contig was determined with CheckM [17], using default parameters. Contigs having completeness higher than 90% and contamination lower than 10% were considered a ‘‘complete” metagenomic assembly. Taxonomic assignments of complete contigs were conducted using GTDB-Tk v1.6.0 [18], based on the Genome taxonomy database [19]. GTDB-Tk uses a combination of metrics, including average nucleotide identity to reference genomes in the NCBI Assembly database, placement in the GTDB reference tree, and the relative evolutionary divergence.

### Genome annotation of novel *Mycoplasma*

For the complete bacterial genome, gene and subsystem annotation was conducted using RAST v2.0 [20]. RNAs were annotated using RNAmmer v1.2 [21]. The Kyoto Encyclopedia of Genes and Genomes (KEGG- BlastKOALA) v2.2 [22] was used to predict biological pathways present in the genome. Ori-Finder 1 [23, 24] was used to identify the Origin of Replication (OriC). Clusters of orthologous genes (COGs) were annotated using eggnog-mapper v2.1.7 [25]. The genome was scanned for virulence factors using the BLAST search tool of the Virulence Factor Database (VFDB) [26] (database used–core and full dataset protein sequence). CRISPRCasFinder v1.1.2 [27] was used to validate predicted CRISPR/CAS systems. The average nucleotide identity (ANI) value was calculated using the Ezbio ANI calculator tool [28], and the average amino acid identity (AAI) was calculated using the webserver available through the Georgia Institute of Technology [29]. The dDDH calculator from Type Strain Genome Server (TYGS) [30] was used to predict digital DNA:DNA hybridization (dDDH) values from intergenomic distances for *Ca*. M. mahonii and its most closely related type strains genome sequences as implied in the Genome to Genome distance calculator (GGDC) [31].

### Screening for *Mycoplasma* from multiple *Gorgonocephalus* individuals

Fifteen *Gorgonocephalus* individuals were screened for *Mycoplasma*. Nine *G*. *chilensis* from Argentinian waters were obtained during the LMG-0605 cruise in 2006 and 6 *G*. *eucnemis* were obtained in 2014 near the University of Washington’s Friday Harbor laboratories in the Northeast Pacific; S1 Table. All samples were collected by KMH by trawl and subsequently stored in a -80°C freezer. Total genomic DNA was extracted using the Qiagen DNeasy blood and tissue kit (Maryland, USA) following the manufacturer’s instructions except that lysis was done overnight to ensure complete digestion due to the calcium carbonate in the arm tissue. Agarose gel electrophoresis was used to verify the integrity of the isolated DNA.

A 716bp region of the 16S RNA gene was targeted for PCR amplification using two oligonucleotide primers (Forward- 5’-ACTCCTACGGGAGGCAGCAGTA-3’; Reverse 5’-TGCACCATCTGTCAYTCYGTTAACCTC-3’) that were slightly modified from previously published *Mycoplasma* universal oligopeptide primers [32] to be more specific to *Ca*. M. mahonii. Thermocycling conditions included an initial denaturation at 98°C for 30 sec, followed by 35 cycles of denaturation at 98°C for 10 sec, annealing at 60°C– 62°C for 30 sec, extension at 72°C 30 sec, and a final extension at 72°C for 5 min. Negative controls were employed in PCRs, samples from Argentinian waters and Northeast Pacific samples were handled in the lab on different dates. Amplified PCR products were examined by 1% gel electrophoresis and purified using the Qiagen QIAquick purification kit (Maryland, USA). The purified template was Sanger sequenced by Genewiz (New Jersey, USA), and bidirectional reads were trimmed and verified using Geneious software (v 2021.2.2) [33].

### Phylogenetic analysis

To determine the phylogenetic affiliation of the new organism, two sets of phylogenetic analyses were conducted. One analysis employed a single gene tree based on 16S rRNA data, allowing for greater taxon sampling. The second analysis employed a multilocus tree, albeit with fewer taxa, to overcome the bias that can be caused by single-gene analysis (e.g. lineage sorting and selection pressure) and to provide a more robust representation of genomic data.

All available 16S rRNA sequences from *Mycoplasma* type strain were obtained from Ezbiocloud database [34] and GenBank [35], additionally, Mycoplasmatales bacteria DT_67 and DT_68, as well as 2 Oyster Mollicutes MAGs, were added to sequences obtained from this study to reconstruct the phylogenetic history of the group. *Bacillus subtilis* was used as an outgroup to root the resultant trees based on current understanding of *Mycoplasma* evolutionary relationships [36, 37]. Sequences were aligned using MAFFT v7.475 [38], with default parameters. Maximum likelihood analysis with bootstrap was employed to reconstruct phylogenetic relationships in IQtree v1.6.12 [39] using the following parameters ‘-bb 100000, -nt AUTO,—runs 5’. These parameters were employed for all IQtree phylogenetic analyses in this study. The substitution model used for the 16S rRNA gene-based phylogeny, GTR+F+R10, was selected as the best model by IQtree’s ModelFinder.

For the multilocus phylogenetic analysis, a representative with a sequenced genome from each major clade present in the 16S rRNA tree was selected and its genome screened for the presence of five single-copy housekeeping genes–*recA*, *lepA*, *dnaK*, *ruvB*, and *gmk*. This gene choice was based on the available literature for *Mycoplasma* phylogeny [40–44]. DNA sequences of these 5 housekeeping genes were aligned using the MAFFT option in Geneious (Geneious Prime 2023.0.4, Java version 11.0.15+10, 64-bit) and then concatenated. The multilocus phylogenetic tree of the concatenated alignment was reconstructed by employing maximum likelihood analysis with bootstrap in IQtree v1.6.12. The substitution model used for the multilocus phylogeny, GTR+F+R6, was selected as the best model by IQtree’s ModelFinder. Additionally, tree topologies for the individual genes were also inferred using a MAFFT v7.475 alignment and maximum likelihood in IQtree v1.6.12. A Bayesian Inference analysis was also run for both the 16S rRNA and multilocus tree using MrBayes [45]. The same set of sequences as mentioned earlier was used, and the GTR+I+G model was chosen through JModeLTest2 [46]. on CRIPES Science Gateway (https://www.phylo.org/index.php/ (accessed on April 2023)), and with 1000000 generations sampled every 500 generations. Burninfrac was set to 0.25. All phylogenetic trees were visualized and edited using Figtree v1.4.4 [47].

### Ethics statement

All fieldwork was carried out under the auspices of the USA Antarctic program or the University of Washington’s Friday Harbor Laboratories, with all applicable rules and permitting requirements followed.

## Results

### Microbial identification and classification

BLAST results of the *G*. *chilensis* assembly against NCBI 16S rRNA genes revealed 4 contigs that contained bacterial 16S rRNA gene fragments. Three contigs were classified as *Mycoplasma* while one was unclassified by the Kraken2 silva database. The 3 classified contigs were 51 Kb, 73 Kb, and 796 Kb in size respectively, 16S rRNA gene percentage identities of the 769kb contig compared to the other contigs ranged from 75% - 88%. Previously described *Mycoplasma* spp. genome sizes range from 540 Kb to 1300 Kb, hence we hypothesized that the 51 Kb and 73 Kb contig were likely to be incomplete or fragments of *Mycoplasma* genomes. CheckM analyses confirmed this interpretation as completeness of 5.72%, 7.14%, and 97.93% were reported for the 73 Kb, 51 Kb, and 796 Kb contigs respectively. In addition, CheckM only reported 0.38% contamination for the 796 Kb contig. Hence, we considered the 796 Kb contig to represent a complete genome, and further downstream analyses were conducted only on this MAG.

GTDB-Tk robustly placed this complete MAG in the order Mycoplasmatales and family Metamycoplasmataceae (GTDB-tk RED value of 0.93; S2 Table) and phylogenetic analysis shows it to be related to a marine clade of *Mycoplasma*. This identified novel *Mycoplasma* genome was designated as “*Candidatus* Mycoplasma mahonii” (formal description given below).

### Genome annotation

#### General features of the genome

**Fig 1:**
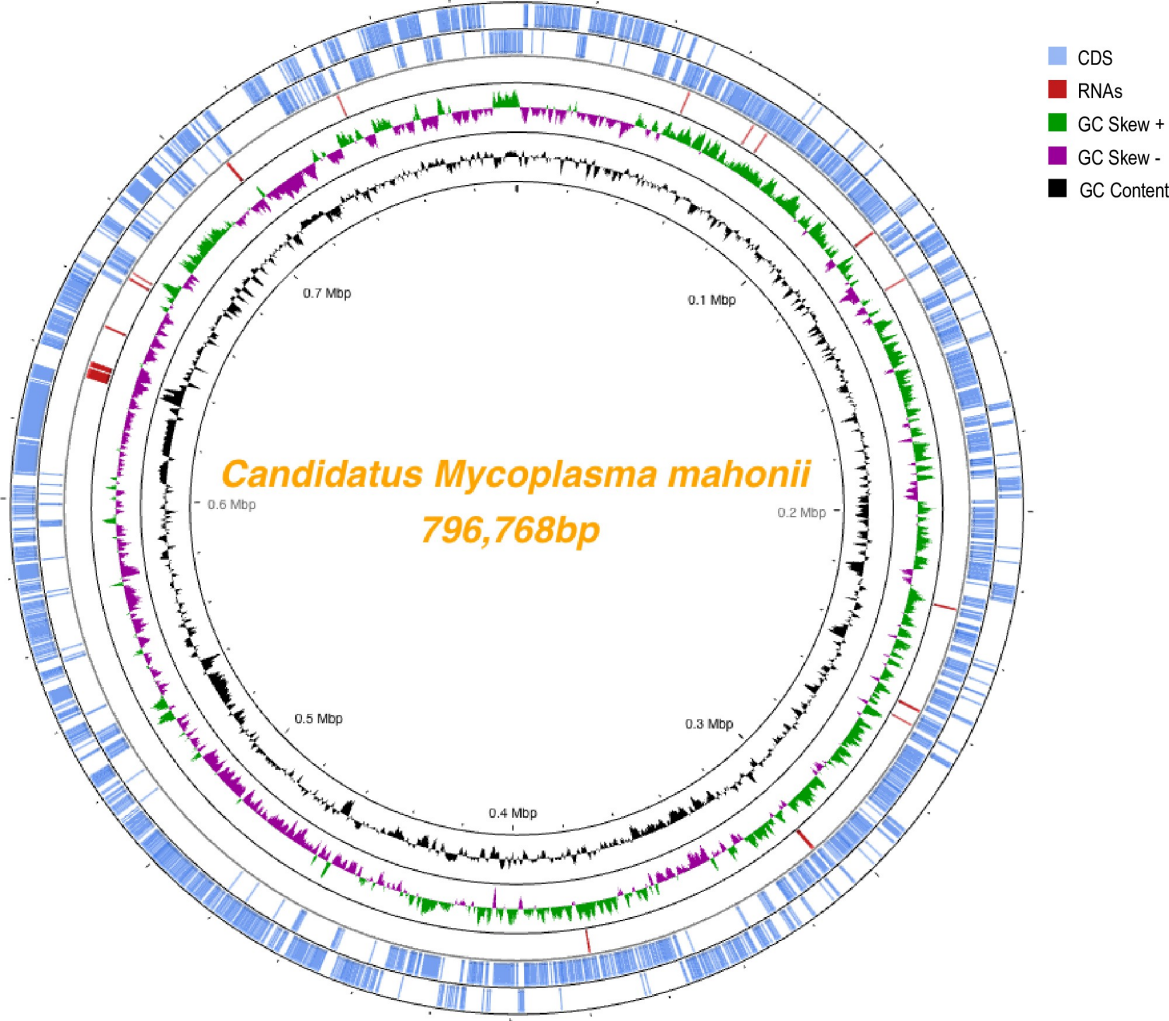
Chromosome atlas of *Candidatus* Mycoplasma mahonii. The scale is shown by the inner black circle. Starting with the outermost rings, the 1st and 2nd circles show predicted coding sequences on the minus and plus strands respectively. The 3rd circle represents RNAs including both tRNAs and rRNAs. The 4th circle represents GC skew (G−C)/(G+C) (green-above mean, purple-below mean; mean = 0.5) and the 5th circle represents mean-centered G+C content of the genome. The figure was generated using Proksee.

**Fig 2:**
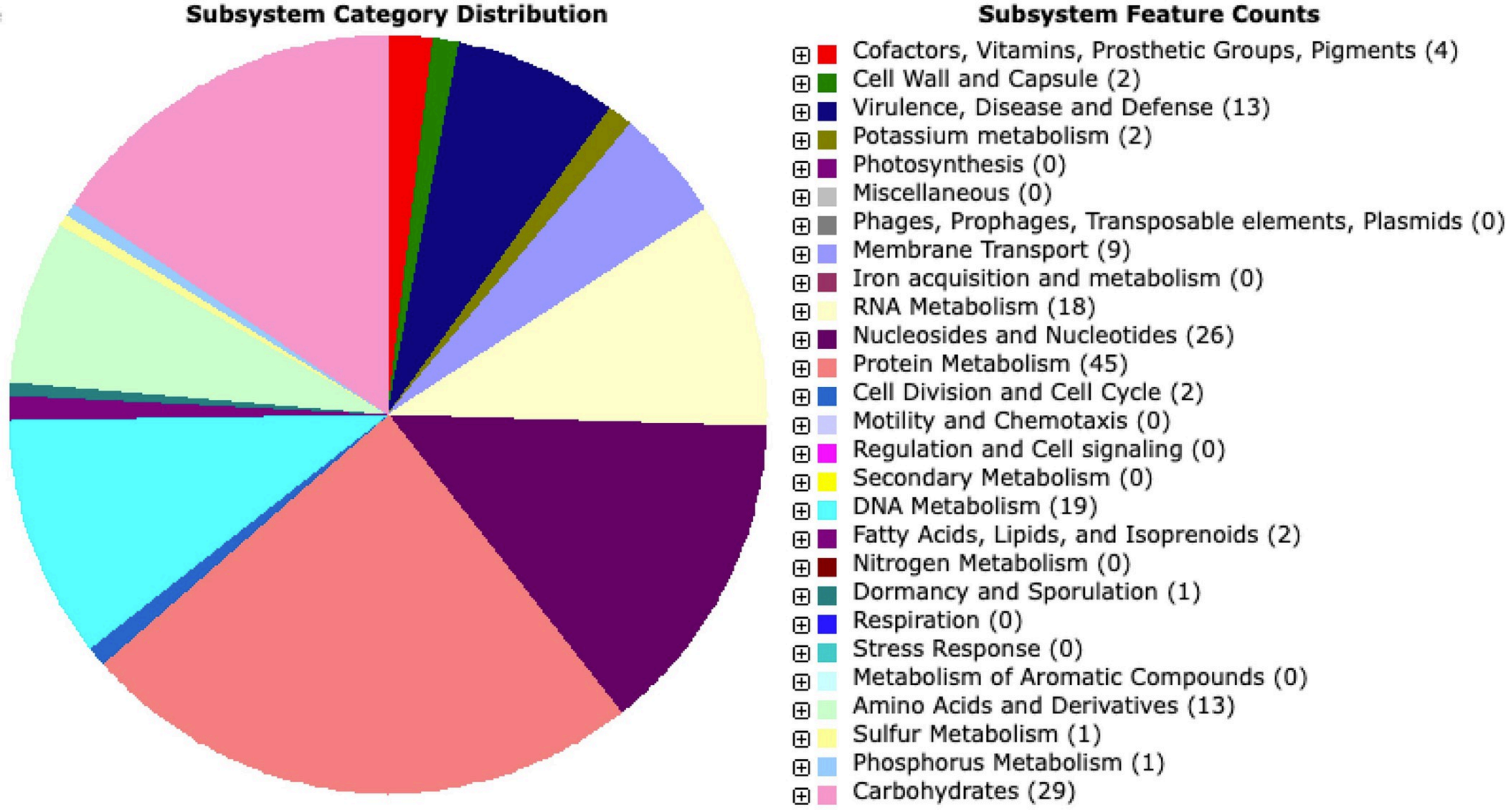
RAST subsystem feature categories. The chart is color-coded based on the corresponding subsystem category.

**Fig 3:**
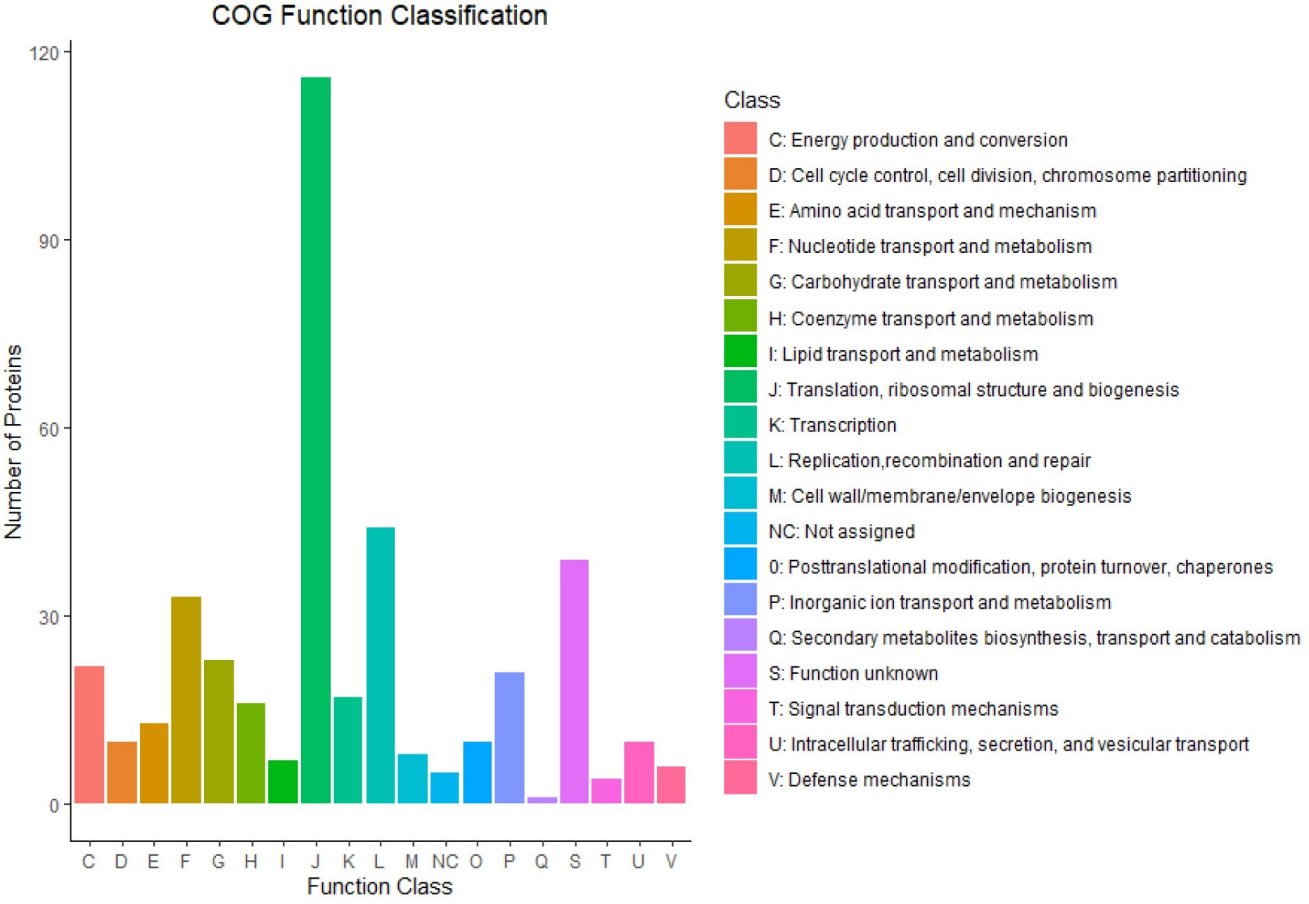
Clusters of orthologous group function classification. The Y-axis represents the number of protein/unigenes belonging to a particular category while the X-axis represents the COG categories.

The novel *Ca*. M. mahonii genome consists of a single chromosome of 796,768 bp with a GC content of 30.1% (Fig 1). The 16S, 23S, and 5S rRNA genes were present as single copies, with the 16S and 23S rRNA genes located in the same operon and the 5S rRNA gene in a separate genomic region. Thirty-one transfer RNAs (tRNA) were identified, and all standard amino acids were represented. RAST predicted a total of 780 protein-coding sequences (CDS) of which 406 CDS (52.1%) were assigned putative functions and 374 CDS (47.9%) were annotated as hypothetical proteins. Repeats comprised 6.9% of the genome. Average gene length of predicted CDS was 887bp. Among the predicted CDS, 397 CDS (50.8%) were classified into Clusters of Orthologous (COG) families comprising 18 functional categories with most genes belonging to the J class (Translational, ribosomal structure, and biogenesis). RAST and eggNOG (COG) annotation were similar to that seen in other *Mycoplasma* spp. genomes based on RAST subsystems (Fig 2 and S3 Table) and COG classifications (Fig 3 and S4 Table). Additionally, RAST subsystem category gene counts were compared between *Ca*. M. mahonii and closely related species (S1 Fig).

To verify the functional abilities of the recovered MAG (i.e., the proposed genome), we examined a series of molecular and cellular pathways and structures, and below we describe select major systems in turn.

#### Replication, transcription, and translation

For DNA replication in *Mycoplasma*, the OriC usually contains *dnaA* boxes and is generally around the vicinity of the *dnaA* gene [48–51]. Using previously published *dnaA* box motif consensus sequence 5’-TTATCCACA-3’ [48, 52], and allowing one mismatch, two putative replication origins were found in the area surrounding *dnaA* gene using Ori-Finder. Both regions possessed the typical features of OriC in prokaryotes (i.e. *dnaA* box, *dnaA* gene vicinity, GC skew inversion) [53].

A total of 43 genes (S5 Table) were predicted to be involved in replication, recombination, and repair. DNA repair appears to be mainly executed by nucleotide excision repair, SOS repair system, and recombination repair. No mismatch-repair system genes (MutHLS) were found.

For transcription, a total of 11 genes were predicted to be involved (S6 Table). Transcription termination and elongation are regulated by *Nus A*, *B*, *G*, and *GreA* genes in this organism. *GreA* prevents transcription arrest while the Nus proteins can induce transcription pausing or stimulate anti-termination [54]. In addition, two transcription factors (*HigA* and *MraZ*) and one heat-inducible transcription repressor gene (*HrcA*) were identified.

Additionally, a total of 126 genes were predicted to be involved in translation, ribosomal structure, and biogenesis including 49 ribosomal proteins, 24 aminoacyl tRNA synthase genes, and 11 translation factors (S7 Table).

#### Secretion system and transporters

The transporter system of *Ca*. M. mahonii consists of 39 genes, which are mainly made up of the ATP-binding cassette (ABC) transporter system and the phosphotransferase (PTS) system (S8 Table). For the PTS system, 4 genes that encode proteins required by the PTS system were present; *pts1* which encodes Enzyme 1 (E1), *ptsH* which encodes Phosphocarrier protein Hpr, *fruA* which encodes a fructose-specific II component (EIIBC or EIIC) and *celA* which encodes a cellobiose-specific II component (EIIB).

Genes encoded by the ABC transport system include 12 ATP-binding proteins, 11 permease proteins, and 2 substrate-binding proteins. Three complete ABC-type transport systems including a phosphate transporter, a phosphonate transporter, and a general nucleoside transporter were present, while others such as oligopeptide transporter, energy-coupling factor transporter, saccharide/lipid transporter, etc. were incomplete. Other genes that were not part of the PTS system or the ABC transport system were associated with other transporters such as magnesium transporter, riboflavin transporter, potassium uptake system proteins, cytosine permease, and an adenine/guanine/hypoxanthine permease.

Genes such as *secA*, *secY*, *secD/F*, *secE*, *secG*, *ffh*, *yidC*, and *FtsY*, which make up the core proteins required for the Sec-SRP secretion pathway (SEC system) and two other genes associated with the Type II secretion system (*comEB* and *comEC*) were present as part of the organism’s bacterial secretion system (S9 Table).

#### Metabolism

According to the annotation data, *Ca*. M. mahonii encode all needed enzymes in the Embden-Meyerhof-Parnas (EMP or glycolysis) pathway, arginine deaminase pathway, F1-ATP synthase, PRPP (phosphoribosyl-pyrophosphate) production, and pyruvate oxidation. Enzymes associated with lipid metabolism in this organism suggest that they utilize glycerol to generate phospholipids (cardiolipin), the only membrane component identified in this organism.

Genes involved in de novo nucleotide synthesis were lacking in *Ca*. M. mahonii; however, this bacterium was predicted to encode nucleotide salvage pathways genes such as *nrdA* and CTP synthase. *Ca*. M. mahonii lacks genes predicted to be required for different intermediate pathways such as the TCA cycle, citric acid cycle and the oxidative phase of the pentose phosphate pathway. Partial pathways for CoA biosynthesis (specifically pantothenate to CoA), phospholipid biosynthesis, riboflavin/FMN/FAD biosynthesis, Tetrahydrofolate (THF) biosynthesis amongst others were present within the *Ca*. M. mahonii genome.

#### Defense system

Two bacterial defense systems to ward off phage infection, including a Type II Restriction—modification (R-M) system and a CRISPR-CAS system are present in the genome of *Ca*. M. mahonii. The Type II restriction system possessed only genes encoding methylase and methyltransferase enzymes but lacks the sequence-specific endonuclease which is usually responsible for recognizing and cleaving specific DNA sequences. No Type I or Type III R-M genes were found. On the other hand, RAST annotation (and CRISPR finder tools) identified 1 CRISPR array of length 9820bp, 148 spacers, and the direct repeat consensus GTTTAAGAATACACAAGAATGATACCACCCCAAAAC. Additionally, a thioredoxin reductase system which is predicted to provide defense against oxidative stress was also present.

### Screening for Mycoplasmas in *Gorgonocephalus*

Nine out of 15 screened basket star samples (6 *G*. *chilensis* samples from Argentinian waters and 3 *G*. *eucnemis* samples from the Northeast Pacific) had a positive PCR result and these amplicons were Sanger sequenced. However, only 1 *G*. *eucnemis* sample had a good quality read and was the only *G*. *eucnemis* sample used in the phylogenetic analysis. These sequences have been deposited to NCBI under the accession numbers OP995472-OP995479.

### Phylogenetic results

**Fig 4:**
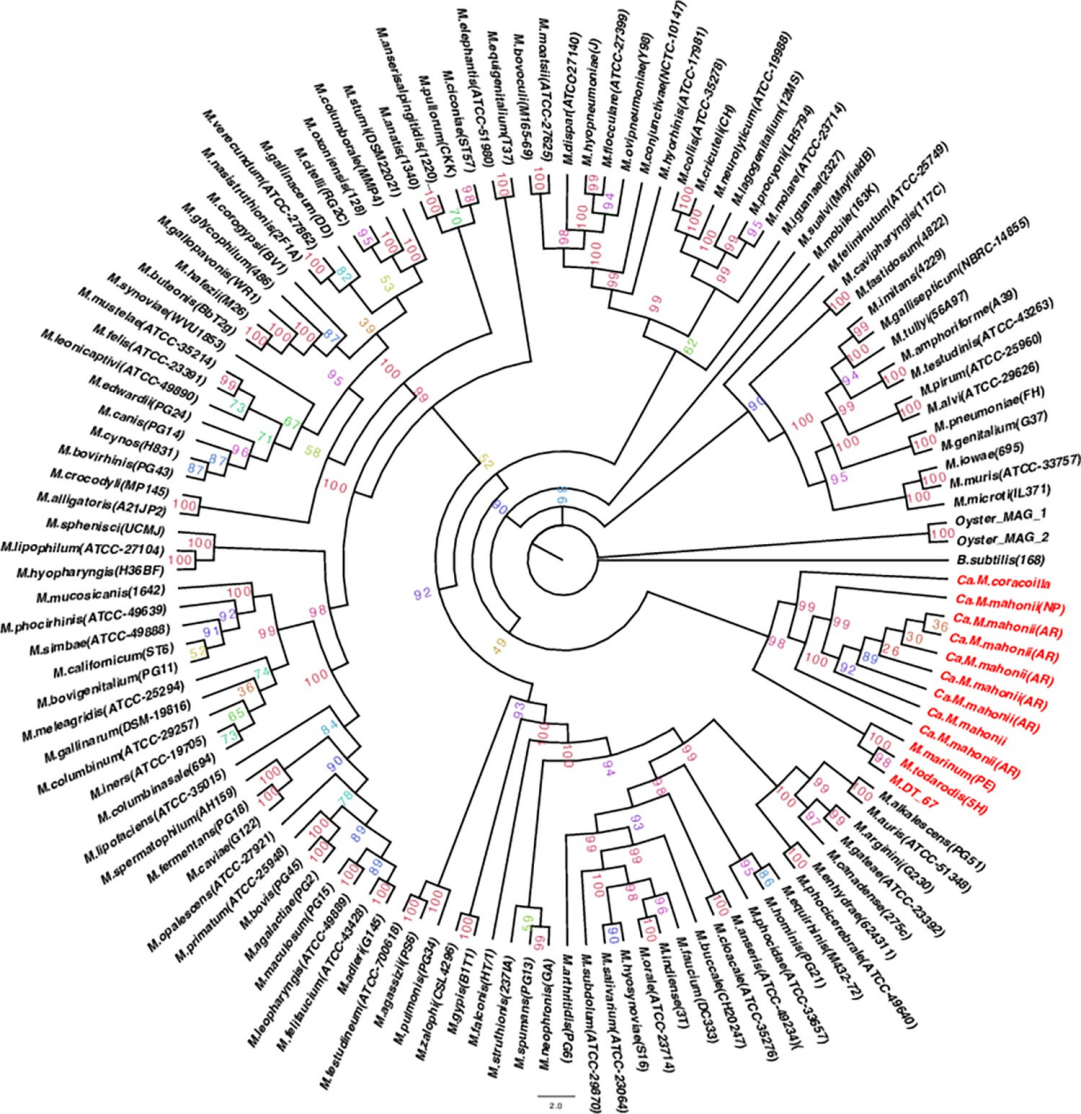
16S rRNA maximum-likelihood phylogenetic tree. The 16S rRNA gene tree was reconstructed based on all available *Mycoplasma* spp. type strain available from the Ezbiocloud and GenBank databases, Mycoplasmatales bacteria DT_67 and DT_68, 2 Oyster Mollicutes MAG and *Ca*. M. mahonii. Bootstrap percentage values are shown on the tree. The tree was generated in IQtree with the GTR+F+R10 model. *Ca*. M. mahonii and other sequences making up the distinct marine clade are shaded red. AR–Argentinian waters samples, NP–North Pacific samples.

**Fig 5:**
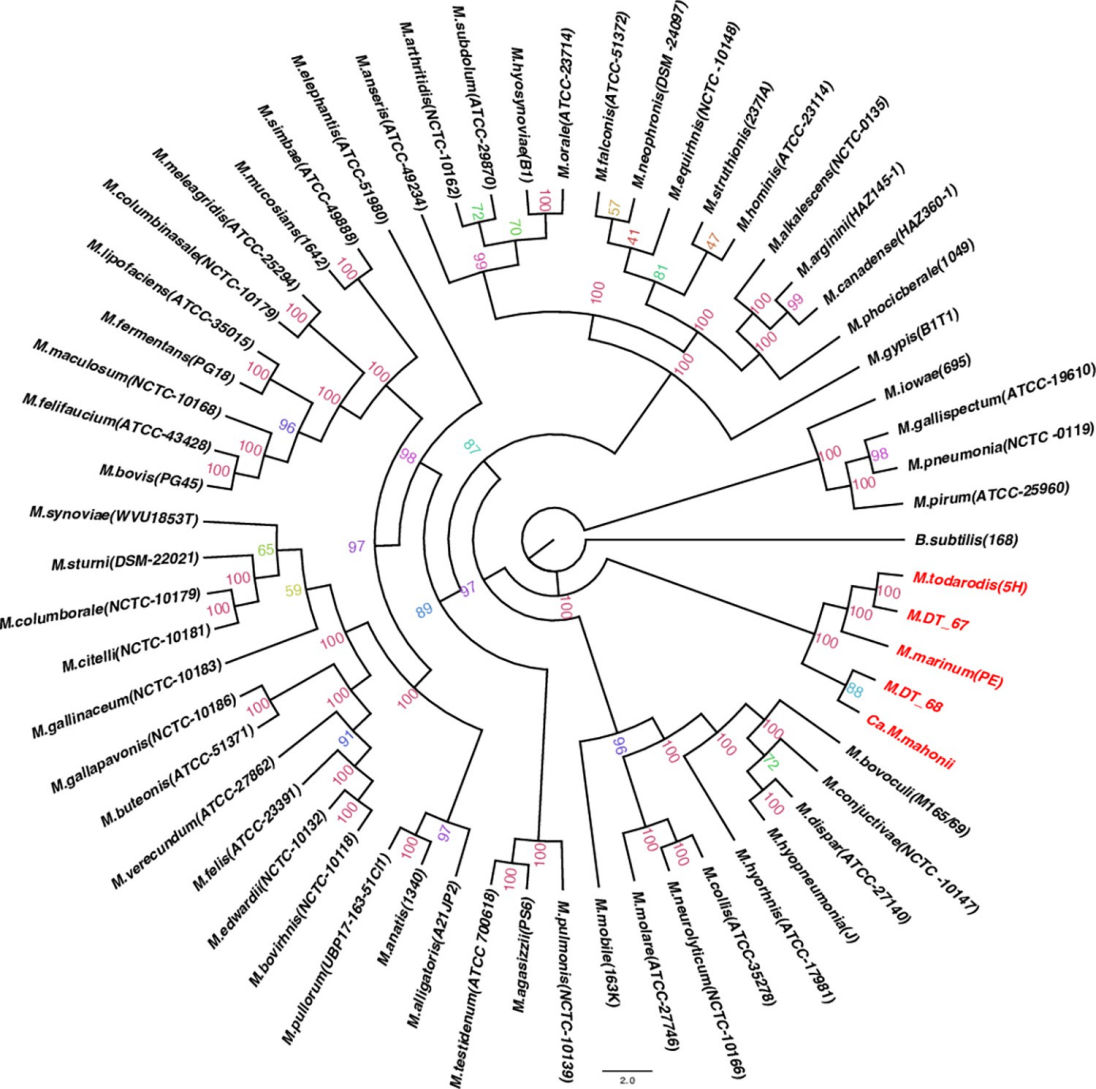
Multilocus maximum-likelihood phylogenetic tree. The multilocus phylogenetic tree was generated based on the concatenated sequence of five single-copy housekeeping genes–*recA*, *lepA*, *dnaK*, *ruvB*, and *gmk*. Bootstrap percentage values are shown on the tree. The tree was generated in IQtree with the GTR+F+R6 model. *Ca*. M. mahonii and other sequences making up the distinct marine clade are shaded red.

16S rRNA gene-based (Fig 4) and multilocus (Fig 5) phylogenetic analyses were used to validate the GTDB-Tk taxonomical assignment and provide higher resolution phylogenetic placement. The 16S rRNA gene analysis consisted of 129 sequences and the multilocus tree consisted of 61 terminals, including sequences from this study (S11 and S12 Tables). Both analyses produced congruent results. *Ca*. M. mahonii was placed within the *Mycoplasma* genus, within a recently characterized lineage of marine taxa, consisting of culturable type strains of *M*. *marinum* PE^T^ (isolated from *Octopus vulgaris*) and *M*. *todarodis* 5H^T^ (isolated from the squid *Todarodes sagittatus*) [10] based on the multilocus tree. Additionally, 2 marine metagenomes, M. DT_67 and M. DT_68 which were isolated from ocean water sinking particles collected at abyssal depths in the Nort Pacific Subtropical Gyre were also part of the marine clade. The phylogenetic tree for each gene used in the multilocus sequence analysis is shown in the S2–S6 Figs. The 16S rRNA topology placed *Ca*. M. mahonii with these same taxa plus *Candidatus* Mycoplasma corallicola (which was not included in the multilocus tree due to an incomplete genome). Furthermore, the *Ca*. M. mahonii 16S rRNA sequences from both Argentinian waters and North Pacific were ∼99.7% identical and formed a monophyletic clade on the 16S rRNA gene-based phylogenetic tree. The Bayesian analysis also showed similar results. *Ca*. M. mahonii formed a monophyletic clade with *Ca*. M. corallicola, M. DT_67, *M*. *marinum*, and *M*. *todarodis* in the 16S rRNA-based phylogenetic tree and also formed a monophyletic clade with M. DT_67, M. DT_68, *M*. *marinum*, and *M*. *todarodis* in the multilocus tree (S7 and S8 Figs). M. DT_68 lacks a 16S rRNA gene and hence was absent in the 16S rRNA phylogenetic tree.

Additionally, 16S sequence similarity analysis using TrueBac ID from Ezbiocloud [55] indicated that *Ca*. M. mahonii was most similar to *Ca*. M. corallicola (89.41%), *M*. *todarodis* (89.1%) and M. *marinum* (88.9%). TrueBac ID also indicated high similarity to *M*. *mobile* (88.9%). However, all the phylogenetic analyses conducted showed M. mobile as an unstable branch as its position varied on the different trees. Consequently, *M*. *mobile* was not further considered as a closely related species. Genomic features of *Candidatus*. M. mahonii and closely related taxa are shown in Table 1.

**Table 1:** Genomic features of *Candidatus* Mycoplasma mahonii and closely related taxa.

	*Candidatus* Mycoplasma mahonii	*M*. *todarodis*	*M*. *marinum*
Genome completeness	97.9%	85%	97.1%
Genome size (bp)	796,768	1,007,879	1,171,149
Number of contigs	1	84	128
%GC	30.1%	30.95%	28.41%
Number of CDS	780	914	1003
Hypothetical genes	374	296	328
16S rRNA gene	1	1	1
23S rRNA gene	1	1	1
5S rRNA gene	1	2	2
Number of tRNAs	31	39	42

**Fig 6:**
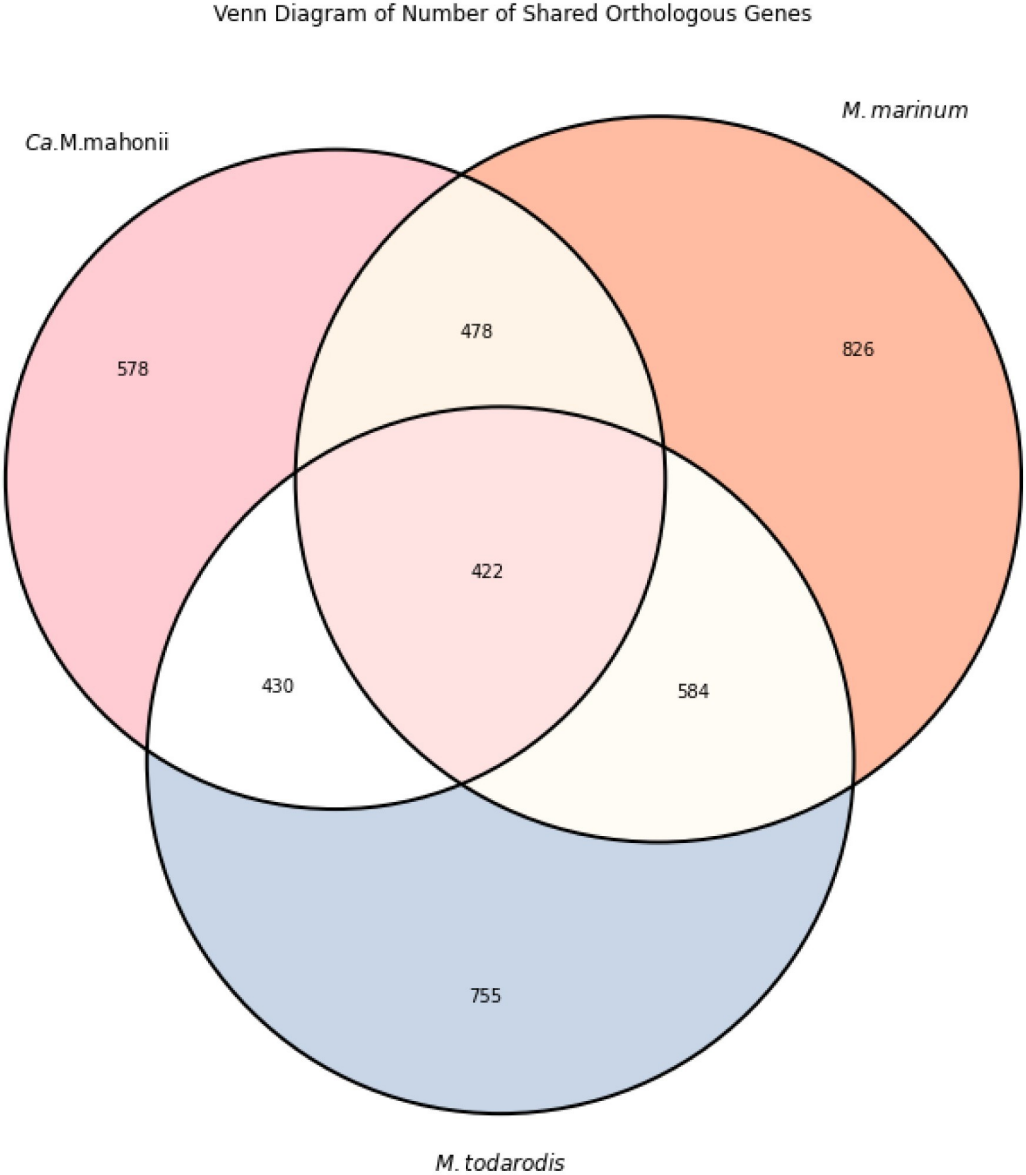
Venn diagram showing the number of shared orthologous genetic groups (OGs) between *Ca*. M. mahonii, *M*. *marinum* and *M*. *todarodis*.

ANI similarity values for *Ca*. M. mahonii and its closely related species were 68.2% (*M*. *marinum*) and 67.4% (*M*. *todarodis*), while the AAI was 50.3% (*M*. *marinum*) and 49.25% (*M*. *todarodis*). A total of 422 orthologous gene groups are shared between *Ca*. M. mahonii, *M*. *todarodis*, and *M*. *marinum* (Fig 6). In addition, the dDDH scores from TYGS pairwise comparison of *Ca*. M. mahonii against identified close strains were less than 70% (~21.5% - 27.9%) indicating that *Ca*. M. mahonii is indeed a novel species (S10 Table).

### Description of “*Candidatus* Mycoplasma mahonii”

The category “Candidatus” is used to describe prokaryotic entities for which information other than just a DNA sequence is available but lacks other characteristics required for description according to the International Code of Nomenclature of Bacteria [56]. The *Mycoplasma* genome described here represents a novel species of *Mycoplasma* and is currently the only representative of this candidate species. The species is designated “*Candidatus* Mycoplasma mahonii” (N.L. gen. masc. n. *mahonii*, of Mahon, named in honor of long-time Antarctic collaborator Andrew Mahon). This species was isolated from *Gorgonocephalus chilensis* (collected May 2006, latitude; -54°49, longitude -60°16, depth 110m) and assignment to “*Candidatus* Mycoplasma mahonii” is based on (i) the associated 16S rRNA gene sequence; accession number—OP995479), (ii) Similarity index score (ANI) of <95% to closest relatives [7, 10], (iii) 97.9% genome completion (according to CheckM analysis), (iv) primer sequence complementary to a region of 16S rRNA- 5’-ACTCCTACGGGAGGCAGCAGTA-3’.

Genome size was ~796 Kb with a G+C content of 30.1%. Within the genome, single copies of rRNA genes and 31 tRNA genes were identified. KEGG-based analysis identified the presence of the following pathways Embden-Meyerhof-Parnas pathway, F1-type ATP Synthase, Acetate production from acetyl-CoA, Folate (vitamin B9) biosynthesis predicted from 7,8-dihydrofolate, nucleotide sugar biosynthesis pathway amongst others. Additionally, KEGG identified both PTS and ABC transport system genes. The genome sequence can be found under NCBI BioSample ID- SAMN32235174 and Accession ID–CP114583.

## Discussion

A novel *Mycoplasma* species, *Candidatus* Mycoplasma mahonii associated with the basket sea star *Gorgonocephalus chilensis* inhabiting the Argentinian waters, was discovered. This taxon also occurs in *Gorgonocephalus* samples from the North Pacific. Phylogenetically, it is part of a recently characterized clade of non-free-living marine lineage of mollicutes that use marine invertebrate organisms as hosts.

The metagenomic assembly of *Ca*. M. mahonii consists of a single 796,768bp contig with a total of 780 predicted protein-coding sequences (CDS). Genomic features of *Ca*. M. mahonii are comparable to previously described *Mycoplasma* species in several respects: 1) The number of predicted CDS in *Ca*. M. mahonii is comparable to other *Mycoplasma* spp., with 635 in *M*. *mobile* [57], 677 in *M*. *pneumonia* [58], and 742 in *M*. *gallisepticum* [51]; 2) The arrangement of rRNA genes is similar to those found in the genome of *M*. *mobile* [57] and *M*. *pulmonis* [59]; 3) Although the exact OriC could not be determined, *Ca*. M. mahonii possesses a tandem arrangement of *dnaA* and *dnaN* genes around the OriC as seen in other mycoplasmas [49, 60, 61]; however, in the case of *Ca*. M. mahonii, ribosomal protein L34 (*rmpH* gene) was also located upstream of the *dnaA* gene in an opposite direction; and 4) The Peptide Release Factor (RF-1) which recognizes the stop codons UAA and UAG and terminates translation was present in *Ca*. M. mahonii, although RF-2 which identifies the UGA stop codon was absent suggesting that this stop codon codes for a protein as seen in other mycoplasmas [62].

Due to its reduced genome size, *Ca*. M. mahonii lacks genes involved in de-novo biosynthesis of nucleotide, lipids, co-factors, and intermediate energy metabolism pathways such as the TCA cycle, citric acid cycle, phosphate pathway, etc., imposing a host-dependent lifestyle on this organism. However, this bacterium is predicted to encode genes involved in the nucleotide salvage pathway such as *nrdA* which converts ribonucleotides to deoxyribonucleotides, and genes involved in nucleotide interconversion such as CTP synthase which converts UTP to CTP as well as permease for pyrimidine and purine transport which allows them to take up these molecules.

Most transport protein-encoding genes in *Ca*. M. mahonii are associated with ABC transport which transports a range of molecules such as peptides, lipids, phosphate, ions, iron, etc., and PTS transport system which transports extracellular sugars such as mannose, fructose, and cellobiose. The presence of these broad substrate transport systems compensates for the lack of various other transport systems such as GLUT (glucose transporters) and may allow the microorganism to obtain nutrients directly from its host rather than synthesizing de-novo, a trend common in mycoplasmas [63]. Additionally, a gene encoding TrkA which is responsible for potassium uptake and is necessary for intracellular survival in prokaryotes was predicted to be present in the genome of *Ca*. M. mahonii.

The lack of a complete TCA cycle, quinones, or cytochromes rules out the possibility of ATP generation through oxidative phosphorylation in this bacterium. Metabolic pathways present in *Ca*. M. mahonii suggest that they are glycolytic species that rely on energy generation through fermentation of sugars, ATP-synthase, pyruvate oxidation to acetate, and hydrolysis of arginine. Additionally, in the non-oxidative phase of the pentose phosphate pathway present in *Ca*. M. mahonii, transaldolase (which catalyzes the transfer of a dihydroxyacetone group from donor compounds to aldehyde acceptor compounds) is absent. This reaction is presumably carried out by an unrecognized protein as the pentose pathway has been reported in other mycoplasmas to be incomplete but functional [57, 64]. In the case of *Ca*. M. mahonii, this reaction is likely carried out by the non-phosphorylating glyceraldehyde-3-phosphate dehydrogenase (GAPN) enzyme, as the gene encoding this enzyme was predicted in the annotation. GAPN reduces NADP to NADPH and can maintain NADPH production in bacteria lacking some pentose phosphate enzyme [65].

The defense systems present in *Ca*. M. mahonii includes R-M Type II system, CRISPR/CAS system, and thioredoxin system. The CRISPR/CAS system and R-M system are a natural pathogenic adaptive immune system that protects prokaryotic organisms against invading nucleic acids most especially viruses.[66, 67]. The R-M system is present in almost all mollicutes sequenced so far [66] while the CRISPR/CAS system has been reported in some but not all mollicutes [68]. On the other hand, the thioredoxin system present in *Ca*. M. mahonii protects it from oxidative stress [69] and has been reported in some *Mycoplasma* species such as *M*. *suis* [61], *M*. *bovis* [70], and *M*. *capricolum* [69].

Virulence factors typically associated with *Mycoplasma* such as adhesion proteins [1], ClpC ATPase [60], variable surface lipoproteins (Vsps) [71, 72], capsular polysaccharides [73], were absent in *Ca*. M. mahonii. Moreover, no virulence factor was detected using the BLAST search tool of the VFDB database, suggesting that *Ca*. M. mahonii is potentially a non-pathogenic *Mycoplasma* species. Interestingly, the absence of virulence factors was also observed in other members of the distinct marine clade of mycoplasmas namely *M*. *marinum*, *M*. *todarodis*, and *Ca*. M. corallicola, suggesting that this monophyletic clade of mycoplasmas are commensals and potentially a natural part of its host microbiome.

Lastly, the high percent identities (~99.5%) between the 16S rRNA genes of *Ca*. M. mahonii from *Gorgonocephalus chilensis* found in Argentinian waters and the Northeast Pacific (*Gorgonocephalus eucnemis*), suggests that this species is broadly distributed and likely native to multiple *Gorgonocephalus* host species. The annotation of *Candidatus* Mycoplasma mahonii, conducted herein, is the first step to understanding the biology and potential pathogenicity of this bacterium. Future studies will expand on this knowledge by focusing on the metabolic pathway interplay between this species and its basket star host.

## Supporting information

S1 Fig RAST subsystem feature categories gene counts for *Ca*. M. mahonii, *M*. *todarodis* and *M*. *marinum*. (TIF)

S2 Fig *lepA* gene tree. The phylogenetic tree was generated in IQtree with the GTR+F+R6 model, bootstrap percentage values are shown on the tree.
(TIF)

S3 Fig *gmk* gene tree. The phylogenetic tree was generated in IQtree with the GTR+F+R5 model, bootstrap percentage values are shown on the tree.
(TIF)

S4 Fig *recA* gene tree. The phylogenetic tree was generated in IQtree with the GTR+F+R5 model, bootstrap percentage values are shown on the tree.
(TIF)

S5 Fig *ruvB* gene tree. The phylogenetic tree was generated in IQtree with the GTR+F+I+G4 model, bootstrap percentage values are shown on the tree.
(TIF)

S6 Fig *dnaK* gene tree. The phylogenetic tree was generated in IQtree with the GTR+F+R5 model, bootstrap percentage values are shown on the tree.
(TIF)

S7 Fig Multilocus bayesian phylogenetic tree. The tree was generated using MrBayes with the GTR+I+G model chosen by JModelTest2. Probability percentage values are shown on the tree. *Ca*. M. mahonii and other sequences making up the distinct marine clade are shaded red.
(TIF)

S8 Fig 16S rRNA bayesian phylogenetic tree. The tree was generated using MrBayes with the GTR+I+G model chosen by JModelTest2. Probability percentage values are shown on the tree. *Ca*. M. mahonii and other sequences making up the distinct marine clade are shaded red. AR–Argentinian waters samples, NP–North Pacific samples.
(TIF)

S1 Table Gorgonocephalus samples. (XLSX)

S2 Table GTDB-TK placement reference. (XLSX)

S3 Table RAST annotation. (XLSX)

S4 Table eggNOG annotation. (XLSX)

S5 Table Genes involved in replication, recombination, and repair. (XLSX)

S6 Table Genes involved in transcription. (XLSX)

S7 Table Genes involved in translation, ribosomal structure, and biogenesis. (XLSX)

S8 Table Genes involved in transport systems. (XLSX)

S9 Table Genes involved in bacterial secretion system. (XLSX)

S10 Table dDDH score of pairwise comparison of *Ca*. M. mahonii vs best match type strain genomes. (XLSX)

S11 Table GenBank IDs of sequences used for multilocus phylogenetic analysis. (XLSX)

S12 Table Accession numbers of sequences used for 16S rRNA phylogenetic analysis. (XLSX)

## References

[1] Razin S, Yogev D, Naot Y. Molecular Biology and Pathogenicity of Mycoplasmas. Microbiol Mol Biol Rev. 1998;62: 1094–1156. doi: 10.1128/MMBR.62.4.1094-1156.1998 9841667 PMC98941

[2] Rasmussen JA, Villumsen KR, Duchêne DA, Puetz LC, Delmont TO, Sveier H, et al. Genome-resolved metagenomics suggests a mutualistic relationship between *Mycoplasma* and salmonid hosts. Commun Biol. 2021;4:1–10. 33990699 10.1038/s42003-021-02105-1 PMC8121932

[3] Sellyei B, Varga Z, Cech G, Varga Á, Székely C. *Mycoplasma* infections in freshwater carnivorous fishes in Hungary. J Fish Dis. 2021;44:297–304. 33128822 10.1111/jfd.13283

[4] Tattar TA. Diseases of Shade Trees. Revised ed. Academic Press, 1989 pp 57–67.

[5] Bano N, Smith AD, Bennett W, Vasquez L, Hollibaugh JT. Dominance of *Mycoplasma* in the guts of the Long-Jawed Mudsucker, *Gillichthys mirabilis*, from five California salt marshes. Environ Microbiol. 2007;9:2636–41. 17803786 10.1111/j.1462-2920.2007.01381.x

[6] Holben WE, Williams P, Saarinen M, Särkilahti LK, Apajalahti JHA. Phylogenetic Analysis of Intestinal Microflora Indicates a Novel *Mycoplasma* Phylotype in Farmed and Wild Salmon. Microb Ecol. 2002;44:175–85. 12082453 10.1007/s00248-002-1011-6

[7] Neulinger SC, Gärtner A, Järnegren J, Ludvigsen M, Lochte K, Dullo WC. Tissue-associated “*Candidatus* mycoplasma corallicola” and filamentous bacteria on the cold-water coral *Lophelia pertusa* (Scleractinia). Appl Environ Microbiol. 2009;75:1437–44. 19114511 10.1128/AEM.01781-08 PMC2648162

[8] Meziti A, Ramette A, Mente E, Kormas KA. Temporal shifts of the Norway lobster (*Nephrops norvegicus*) gut bacterial communities. FEMS Microbiol Ecol. 2010;74:472–84. 20831590 10.1111/j.1574-6941.2010.00964.x

[9] Iehata S, Valenzuela F, Riquelme C. Analysis of bacterial community and bacterial nutritional enzyme activity associated with the digestive tract of wild Chilean octopus (*Octopus mimus* Gould, 1852). Aquac Res. 2015;46:861–73.

[10] Ramírez AS, Vega-Orellana OM, Viver T, Poveda JB, Rosales RS, Poveda CG, et al. First description of two moderately halophilic and psychrotolerant *Mycoplasma* species isolated from cephalopods and proposal of *Mycoplasma marinum* sp. nov. and *Mycoplasma todarodis* sp. nov. Syst Appl Microbiol. 2019;42:457–67. 31072660 10.1016/j.syapm.2019.04.003

[11] Huang Z Bin, Guo F, Zhao J, Li WD, Ke CH. Molecular analysis of the intestinal bacterial flora in cage-cultured adult small abalone, *Haliotis diversicolor*. Aquac Res. 2010;41:e760–9.

[12] Tanaka R, Ootsubo M, Sawabe T, Ezura Y, Tajima K. Biodiversity and in situ abundance of gut microflora of abalone (*Haliotis discus hannai*) determined by culture-independent techniques. Aquaculture. 2004;241:453–63.

[13] Nakagawa S, Saito H, Tame A, Hirai M, Yamaguchi H, Sunata T, et al. Microbiota in the coelomic fluid of two common coastal starfish species and characterization of an abundant Helicobacter-related taxon. Sci. Rep. 2017;7:1–10. 28821872 10.1038/s41598-017-09355-2 PMC5562702

[14] Cheng H, Concepcion GT, Feng X, Zhang H, Li H. Haplotype-resolved de novo assembly using phased assembly graphs with hifiasm. Nat Methods. 2021;18:170–175. doi: 10.1038/s41592-020-01056-5 33526886 PMC7961889

[15] Sayers EW, Bolton EE, Brister JR, Canese K, Chan J, Comeau DC, et al. Database resources of the National Center for Biotechnology Information. Nucleic Acids Res. 2022;50:D20–D26. doi: 10.1093/nar/gkab1112 34850941 PMC8728269

[16] Wood DE, Lu J, Langmead B. Improved metagenomic analysis with Kraken 2. Genome Biol. 2019;20:257. doi: 10.1186/s13059-019-1891-0 31779668 PMC6883579

[17] Parks DH, Imelfort M, Skennerton CT, Hugenholtz P, Tyson GW. CheckM: assessing the quality of microbial genomes recovered from isolates, single cells, and metagenomes. Genome Res. 2015;25:1043–55. doi: 10.1101/gr.186072.114 25977477 PMC4484387

[18] Chaumeil PA, Mussig AJ, Hugenholtz P, Parks DH. GTDB-Tk: a toolkit to classify genomes with the Genome Taxonomy Database. Bioinformatics. 2020;36:1925–7. 10.1093/bioinformatics/btz848 PMC7703759 31730192

[19] Parks DH, Chuvochina M, Rinke C, Mussig AJ, Chaumeil PA, Hugenholtz P. GTDB: an ongoing census of bacterial and archaeal diversity through a phylogenetically consistent, rank normalized and complete genome-based taxonomy. Nucleic Acids Res. 2022;50:D785–94. 34520557 10.1093/nar/gkab776 PMC8728215

[20] Aziz RK, Bartels D, Best A, DeJongh M, Disz T, Edwards RA, et al. The RAST Server: Rapid annotations using subsystems technology. BMC Genomics. 2008;9:1–15. 18261238 10.1186/1471-2164-9-75 PMC2265698

[21] Lagesen K, Hallin P, Rødland EA, Stærfeldt HH, Rognes T, Ussery DW. RNAmmer: consistent and rapid annotation of ribosomal RNA genes. Nucleic Acids Res. 2007;35:3100–3108. doi: 10.1093/nar/gkm160 17452365 PMC1888812

[22] Kanehisa M, Furumichi M, Sato Y, Ishiguro-Watanabe M, Tanabe M. KEGG: integrating viruses and cellular organisms. Nucleic Acids Res. 2021;49:D545–D551. doi: 10.1093/nar/gkaa970 33125081 PMC7779016

[23] Gao F, Zhang CT. Ori-Finder: A web-based system for finding oriCs in unannotated bacterial genomes. BMC Bioinformatics. 2008;9:1–6. 18237442 10.1186/1471-2105-9-79 PMC2275245

[24] Luo H, Quan CL, Peng C, Gao F. Recent development of Ori-Finder system and DoriC database for microbial replication origins. Brief Bioinform. 2019;20:1114–24. doi: 10.1093/bib/bbx174 29329409

[25] Cantalapiedra CP, Hern Andez-Plaza A, Letunic I, Bork P, Huerta-Cepas J. eggNOG-mapper v2: Functional Annotation, Orthology Assignments, and Domain Prediction at the Metagenomic Scale. Mol Biol Evol. 2021;38:5825–9. doi: 10.1093/molbev/msab293 34597405 PMC8662613

[26] Chen L, Yang J, Yu J, Yao Z, Sun L, Shen Y, et al. VFDB: a reference database for bacterial virulence factors. Nucleic Acids Res. 2005;33:D325–D328. doi: 10.1093/nar/gki008 15608208 PMC539962

[27] Couvin D, Bernheim A, Toffano-Nioche C, Touchon M, Michalik J, Néron B, et al. CRISPRCasFinder, an update of CRISRFinder, includes a portable version, enhanced performance and integrates search for Cas proteins. Nucleic Acids Res. 2018;46:W246–51. doi: 10.1093/nar/gky425 29790974 PMC6030898

[28] Yoon SH, Ha S min, Lim J, Kwon S, Chun J. A large-scale evaluation of algorithms to calculate average nucleotide identity. Antonie van Leeuwenhoek, International Journal of General and Molecular Microbiology. 2017;110:1281–6. doi: 10.1007/s10482-017-0844-4 28204908

[29] Rodriguez-R LM, Konstantinidis KT. The enveomics collection: a toolbox for specialized analyses of microbial genomes and metagenomes. PeerJ Preprints. 2016;4:e1900v1.

[30] Meier-Kolthoff JP, Göker M. TYGS is an automated high-throughput platform for state-of-the-art genome-based taxonomy. Nat Commun. 2019;10:2182. doi: 10.1038/s41467-019-10210-3 31097708 PMC6522516

[31] Meier-Kolthoff JP, Auch AF, Klenk HP, Göker M. Genome sequence-based species delimitation with confidence intervals and improved distance functions. BMC Bioinformatics. 2013;14:1–14. 23432962 10.1186/1471-2105-14-60 PMC3665452

[32] Van Kuppeveld FJM, Van der Logt JTM, Angulo AF, Van Zoest MJ, Quint WGV, Niesters HGM, et al. Genus- and species-specific identification of mycoplasmas by 16S rRNA amplification. Appl Environ Microbiol. 1992;58:2606–2615. doi: 10.1128/aem.58.8.2606-2615.1992 1381174 PMC195828

[33] Kearse M, Moir R, Wilson A, Stones-Havas S, Cheung M, Sturrock S, et al. Geneious Basic: An integrated and extendable desktop software platform for the organization and analysis of sequence data. Bioinformatics. 2012;28:1647–9. doi: 10.1093/bioinformatics/bts199 22543367 PMC3371832

[34] Yoon SH, Ha SM, Kwon S, Lim J, Kim Y, Seo H, et al. Introducing EzBioCloud: a taxonomically united database of 16S rRNA gene sequences and whole-genome assemblies. Int J Syst Evol Microbiol. 2017;67:1613–1617. doi: 10.1099/ijsem.0.001755 28005526 PMC5563544

[35] Sayers EW, Cavanaugh M, Clark K, Ostell J, Pruitt KD, Karsch-Mizrachi I. GenBank. Nucleic Acids Res. 2020;48:D84–6. doi: 10.1093/nar/gkz956 31665464 PMC7145611

[36] Weisburg WG, Tully JG, Rose DL, Petzel JP, Oyaizu H, Weisburg WG;, et al. A Phylogenetic Analysis of the Mycoplasmas: Basis for Their Classification. J Bacteriol. 1989;171:6455–6467. doi: 10.1128/jb.171.12.6455-6467.1989 2592342 PMC210534

[37] Wolf M, Müller T, Dandekar T, Pollack JD. Phylogeny of Firmicutes with special reference to *Mycoplasma* (Mollicutes) as inferred from phosphoglycerate kinase amino acid sequence data. Int J Syst Evol Microbiol. 2004;54:871–5. 15143038 10.1099/ijs.0.02868-0

[38] Katoh K, Standley DM. MAFFT Multiple Sequence Alignment Software Version 7: Improvements in Performance and Usability. Mol Biol Evol. 2013;30:772–780. doi: 10.1093/molbev/mst010 23329690 PMC3603318

[39] Nguyen LT, Schmidt HA, von Haeseler A, Minh BQ. IQ-TREE: A Fast and Effective Stochastic Algorithm for Estimating Maximum-Likelihood Phylogenies. Mol Biol Evol. 2015;32:268–274. doi: 10.1093/molbev/msu300 25371430 PMC4271533

[40] Mayor D, Jores J, Korczak BM, Kuhnert P. Multilocus sequence typing (MLST) of *Mycoplasma hyopneumoniae*: A diverse pathogen with limited clonality. Vet Microbiol. 2008;127:63–72. 17884308 10.1016/j.vetmic.2007.08.010

[41] Manso-Silván L, Dupuy V, Lysnyansky I, Ozdemir U, Thiaucourt F. Phylogeny and molecular typing of *Mycoplasma agalactiae* and *Mycoplasma bovis* by multilocus sequencing. Vet Microbiol. 2012;161:104–12. 22841405 10.1016/j.vetmic.2012.07.015

[42] Tocqueville V, Ferré S, Nguyen NHP, Kempf I, Marois-Créhana C. Multilocus sequence typing of *Mycoplasma hyorhinis* strains identified by a real-time TaqMan PCR assay. J Clin Microbiol. 2014;52:1664–71. 24622092 10.1128/JCM.03437-13 PMC3993664

[43] Dijkman R, Feberwee A, Landman WJM. Development and evaluation of a multi-locus sequence typing scheme for *Mycoplasma synoviae*. Avian Pathology. 2016;45:426–42. 26926568 10.1080/03079457.2016.1154135

[44] Bekő K, Kreizinger Z, Sulyok KM, Kovács ÁB, Grózner D, Catania S, et al. Genotyping *Mycoplasma gallisepticum* by multilocus sequence typing. Vet Microbiol. 2019;231:191–6. 30955809 10.1016/j.vetmic.2019.03.016

[45] Ronquist F, Huelsenbeck JP. MrBayes 3: Bayesian phylogenetic inference under mixed models. Bioinformatics. 2003;19:1572–4. doi: 10.1093/bioinformatics/btg180 12912839

[46] Darriba D, Taboada GL, Doallo R, Posada D. jModelTest 2: more models, new heuristics and high-performance computing. Nat Methods. 2012;9:772. 10.1038/nmeth.2109 PMC4594756 22847109

[47] Rambaut A. Figtree v1.4.4. 2020. Http://TreeBioEdAcUk/Software/Figtree/.

[48] Fujikawa N, Kurumizaka H, Nureki O, Terada T, Shirouzu M, Katayama T, et al. Structural basis of replication origin recognition by the DnaA protein. Nucleic Acids Res. 2003;31:2077–86. doi: 10.1093/nar/gkg309 12682358 PMC153737

[49] Cordova CMM, Lartigue C, Sirand-Pugnet P, Renaudin J, Cunha RAF, Blanchard A. Identification of the origin of replication of the *Mycoplasma pulmonis* chromosome and its use in oriC replicative plasmids. J Bacteriol. 2002;184:5426–35. 12218031 10.1128/JB.184.19.5426-5435.2002 PMC135349

[50] Chopra-Dewasthaly R, Marenda M, Rosengarten R, Jechlinger W, Citti C. Construction of the first shuttle vectors for gene cloning and homologous recombination in *Mycoplasma agalactiae*. FEMS Microbiol Lett. 2005;253:89–94. 16213670 10.1016/j.femsle.2005.09.021 PMC6485543

[51] Papazisi L, Gorton TS, Kutish G, Markham PF, Browning GF, Nguyen DK, et al. The complete genome sequence of the avian pathogen *Mycoplasma gallisepticum* strain R low Microbiology (Reading). 2003;149:2307–2316. 10.1099/mic.0.26427-0 12949158

[52] Messer W. The bacterial replication initiator DnaA. DnaA and oriC, the bacterial mode to initiate DNA replication. FEMS Microbiol Rev. 2002;26:355–74. doi: 10.1111/j.1574-6976.2002.tb00620.x 12413665

[53] Mackiewicz P, Zakrzewska-Czerwińska J, Zawilak A, Dudek MR, Cebrat S. Where does bacterial replication start? Rules for predicting the oriC region. Nucleic Acids Res. 2004;32:3781–91. doi: 10.1093/nar/gkh699 15258248 PMC506792

[54] Borukhov S, Lee J, Laptenko O. Bacterial transcription elongation factors: new insights into molecular mechanism of action. Mol Microbiol. 2005;55:1315–24. doi: 10.1111/j.1365-2958.2004.04481.x 15720542

[55] Ha S-M, Kim CK, Roh J, Byun J-H, Yang S-J, Choi S-B, et al. Application of the Whole Genome-Based Bacterial Identification System, TrueBac ID, Using Clinical Isolates That Were Not Identified With Three Matrix-Assisted Laser Desorption/Ionization Time-of-Flight Mass Spectrometry (MALDI-TOF MS) Systems. Ann Lab Med. 2019;39:530–6. doi: 10.3343/alm.2019.39.6.530 31240880 PMC6660342

[56] Parker CT, Tindall BJ, Garrity GM. International code of nomenclature of Prokaryotes. Int J Syst Evol Microbiol. 2019;69:S1–S111. doi: 10.1099/ijsem.0.000778 26596770

[57] Jaffe JD, Stange-Thomann N, Smith C, DeCaprio D, Fisher S, Butler J, et al. The Complete Genome and Proteome of *Mycoplasma mobile*. Genome Res. 2004;14:1447–61. 15289470 10.1101/gr.2674004 PMC509254

[58] Himmelreich R, Hilbert H, Plagens H, Pirkl E, Li B-C, Herrmann R. Complete sequence analysis of the genome of the bacterium *Mycoplasma pneumoniae*. Nucleic Acids Res. 1996;24:4420–49. 8948633 10.1093/nar/24.22.4420 PMC146264

[59] Chambaud I, Heilig R, Ferris S, Barbe V, Samson D, Galisson F, et al. The complete genome sequence of the murine respiratory pathogen *Mycoplasma pulmonis*. Nucleic Acids Res. 2001;29:2145–2153. 11353084 10.1093/nar/29.10.2145 PMC55444

[60] Chen S, Hao H, Zhao P, Thiaucourt F, He Y, Gao P, et al. Genome-wide analysis of the first sequenced *Mycoplasma capricolum* subsp. *capripneumoniae* strain M1601. G3: Genes, Genomes, Genetics. 2017;7:2899–906. 28754725 10.1534/g3.117.300085 PMC5592918

[61] Guimaraes AMS, Santos AP, SanMiguel P, Walter T, Timenetsky J, Messick JB. Complete Genome Sequence of *Mycoplasma suis* and Insights into Its Biology and Adaption to an Erythrocyte Niche. PLoS One. 2011;6:e19574. 21573007 10.1371/journal.pone.0019574 PMC3091866

[62] Inamine JM, Ho K-C, Loechel S, Hul P-C. Evidence that UGA Is Read as a Tryptophan Codon Rather Than as a Stop Codon by *Mycoplasma pneumoniae*, *Mycoplasma genitalium*, and *Mycoplasma gallisepticum*. J Bacteriol. 1990;172:504–6. 2104612 10.1128/jb.172.1.504-506.1990 PMC208464

[63] Sima Naderi, Saier MH Jr. MicroCorrespondance. Mol Microbiol. 1996;22:389–91.8930922

[64] Yus E, Maier T, Michalodimitrakis K, Van Noort V, Yamada T, Chen WH, et al. Impact of genome reduction on bacterial metabolism and its regulation. Science (1979). 2009;326:1263–8. doi: 10.1126/science.1177263 19965476

[65] Boyd DA, Cvitkovitch DG, Hamilton IR. Sequence, Expression, and Function of the Gene for the Nonphosphorylating, NADP-Dependent Glyceraldehyde-3-Phosphate Dehydrogenase of Streptococcus mutans. J Bacteriol. 1995;177:2622–727. doi: 10.1128/jb.177.10.2622-2627.1995 7751269 PMC176930

[66] Brocchi M, de Vasconcelos ATR, Zaha A. Restriction-modification systems in *Mycoplasma* spp. Genet Mol Biol. 2007;30:236–44.

[67] Hille F, Richter H, Wong SP, Bratovi M, Ressel S, Charpentier E. Leading Edge The Biology of CRISPR-Cas: Backward and Forward. Cell. 2018;172:1239–59. 29522745 10.1016/j.cell.2017.11.032

[68] Ipoutcha T, Tsarmpopoulos I, Talenton V, Gaspin C, Moisan A, Walker CA, et al. Multiple Origins and Specific Evolution of CRISPR/Cas9 Systems in Minimal Bacteria (Mollicutes). Front Microbiol. 2019;10:2701. doi: 10.3389/fmicb.2019.02701 31824468 PMC6882279

[69] Ben-Menachem G, Himmelreich R, Herrmann R, Aharonowitz Y, Rottem S. The thioredoxin reductase system of mycoplasmas. Microbiology (Reading). 1997;143 (Pt 6):1933–40. doi: 10.1099/00221287-143-6-1933 9202470

[70] Li Y, Zheng H, Liu Y, Jiang Y, Xin J, Chen W, et al. The Complete Genome Sequence of *Mycoplasma bovis* Strain Hubei-1. PLoS One. 2011;6:e20999. 21731639 10.1371/journal.pone.0020999 PMC3120828

[71] Bürki S, Frey J, Pilo P. Virulence, persistence and dissemination of *Mycoplasma bovis*. Vet Microbiol. 2015;179:15–22. 25824130 10.1016/j.vetmic.2015.02.024

[72] Seymour LM, Jenkins C, Deutscher AT, Raymond BBA, Padula MP, Tacchi JL, et al. Mhp182 (P102) binds fibronectin and contributes to the recruitment of plasmin(ogen) to the *Mycoplasma hyopneumoniae* cell surface. Cell Microbiol. 2012;14:81–94. 21951786 10.1111/j.1462-5822.2011.01702.x

[73] Pilo P, Vilei EM, Peterhans E, Bonvin-Klotz L, Stoffel MH, Dobbelaere D, et al. A Metabolic Enzyme as a Primary Virulence Factor of *Mycoplasma mycoides* subsp. mycoides Small Colony. J Bacteriol. 2005;187:6824–6831. 16166545 10.1128/JB.187.19.6824-6831.2005 PMC1251598

