# Supplementary figures and images for: Genomic characterization of a novel, widely distributed Mycoplasma species “Candidatus Mycoplasma mahonii” associated with the brittlestar Gorgonocephalus chilensis
Source: PLoS One. 2023 Aug 24;18(8):e0290305. doi: 10.1371/journal.pone.0290305 (PMC10449156; doi:10.1371/journal.pone.0290305)

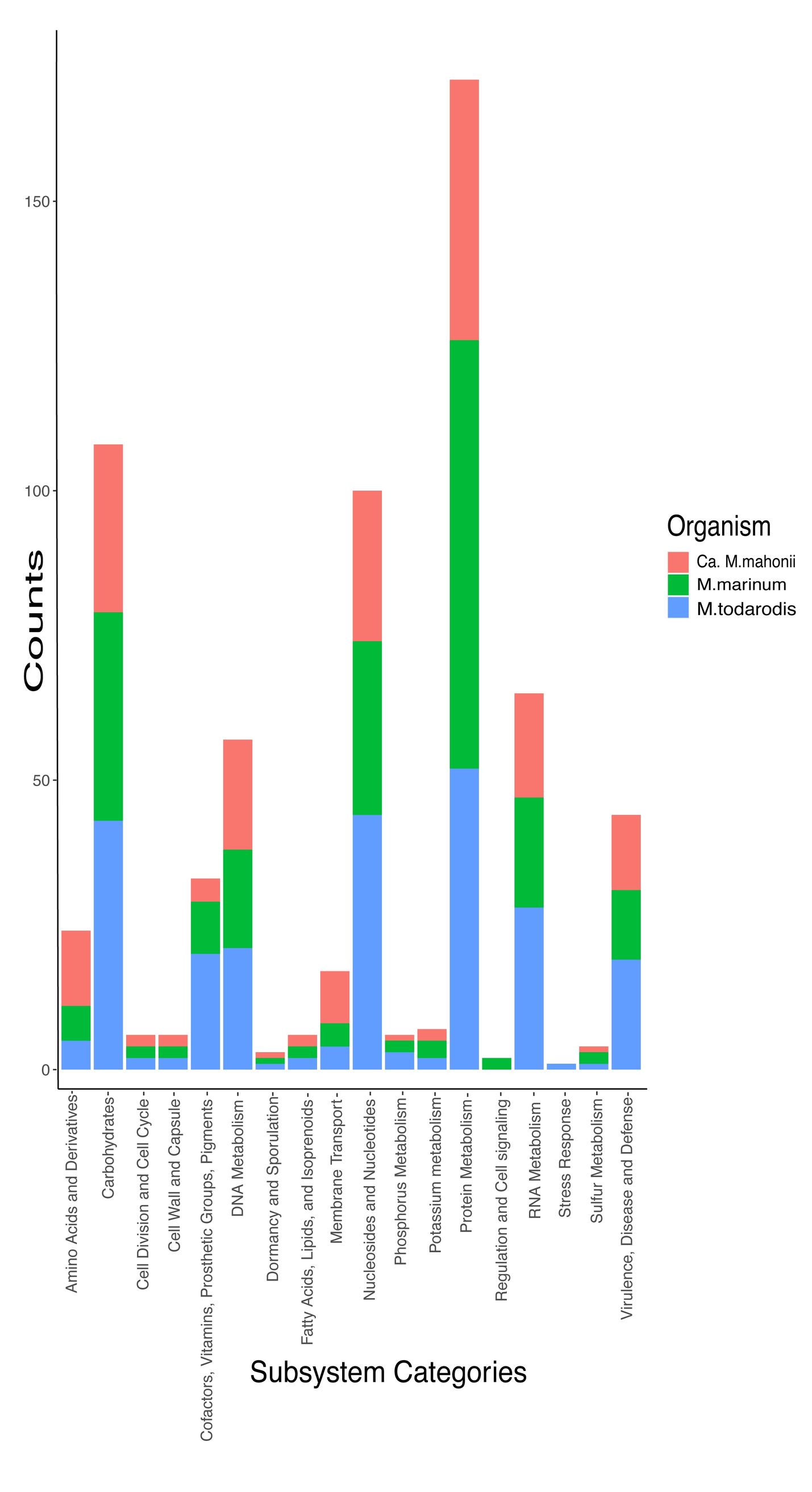

Supplement: S1 Fig — (TIF) [file pone.0290305.s001.tif]

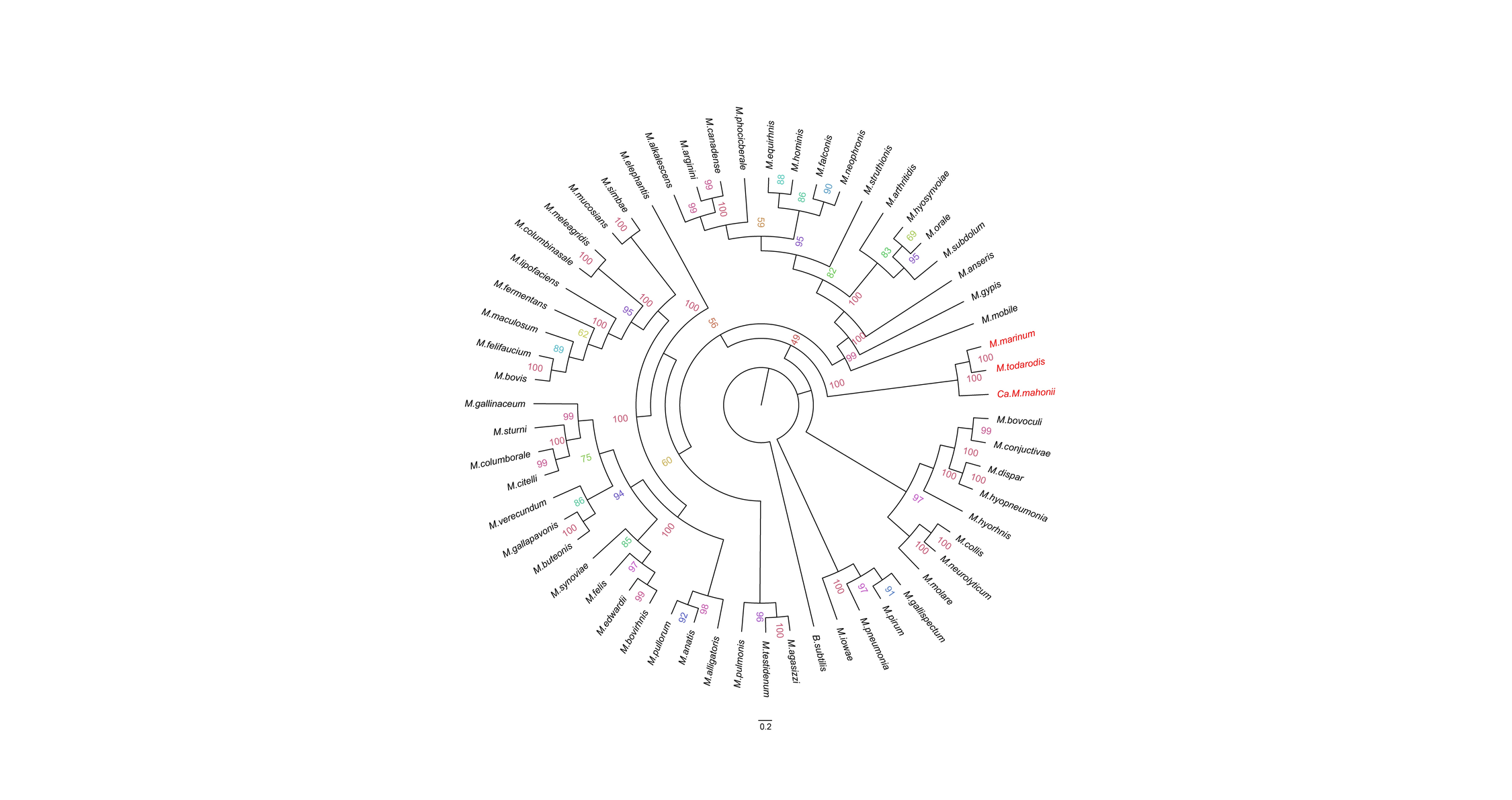

Supplement: S2 Fig — The phylogenetic tree was generated in IQtree with the GTR+F+R6 model, bootstrap percentage values are shown on the tree. (TIF) [file pone.0290305.s002.tif]

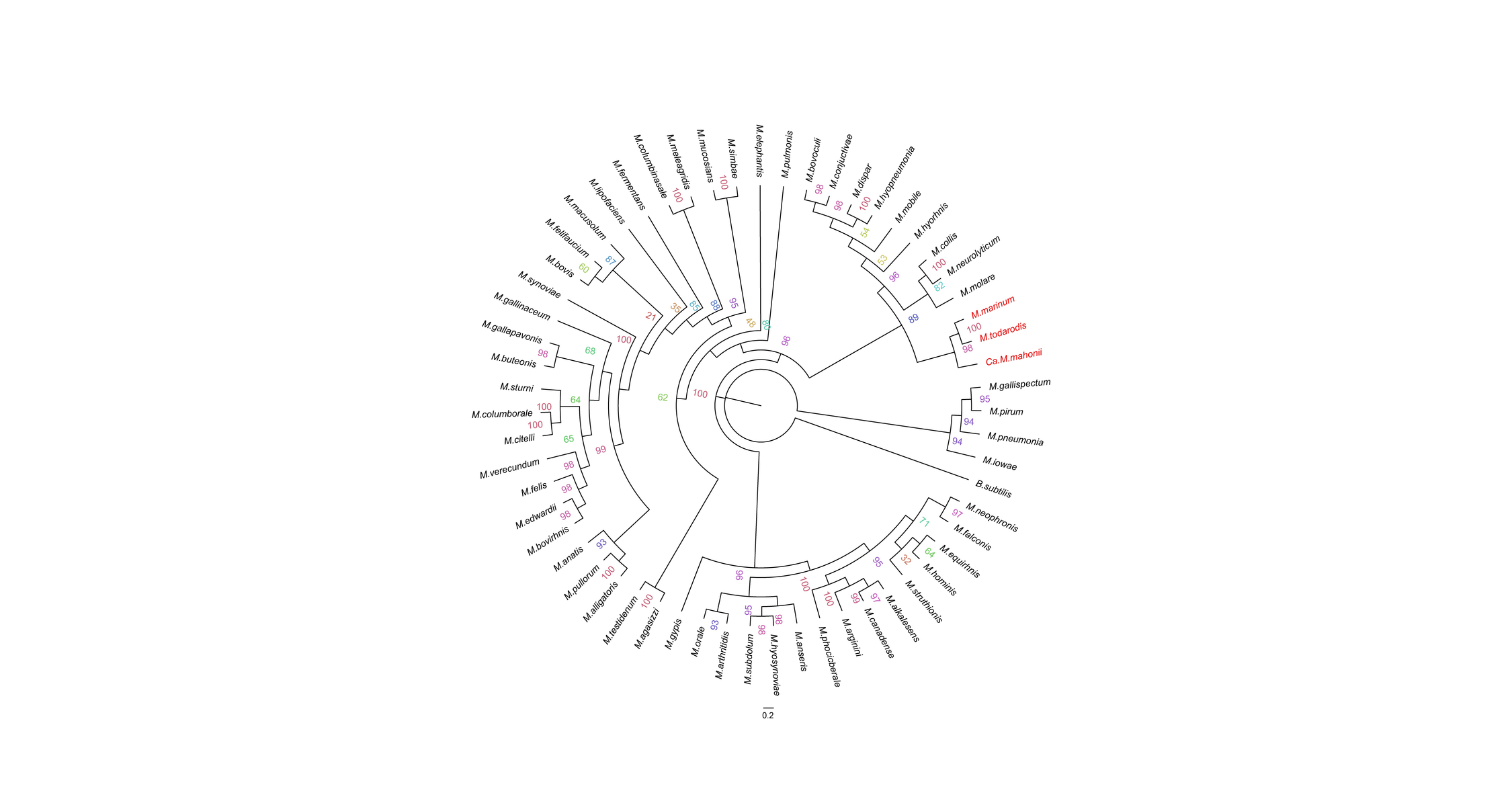

Supplement: S3 Fig — The phylogenetic tree was generated in IQtree with the GTR+F+R5 model, bootstrap percentage values are shown on the tree. (TIF) [file pone.0290305.s003.tif]

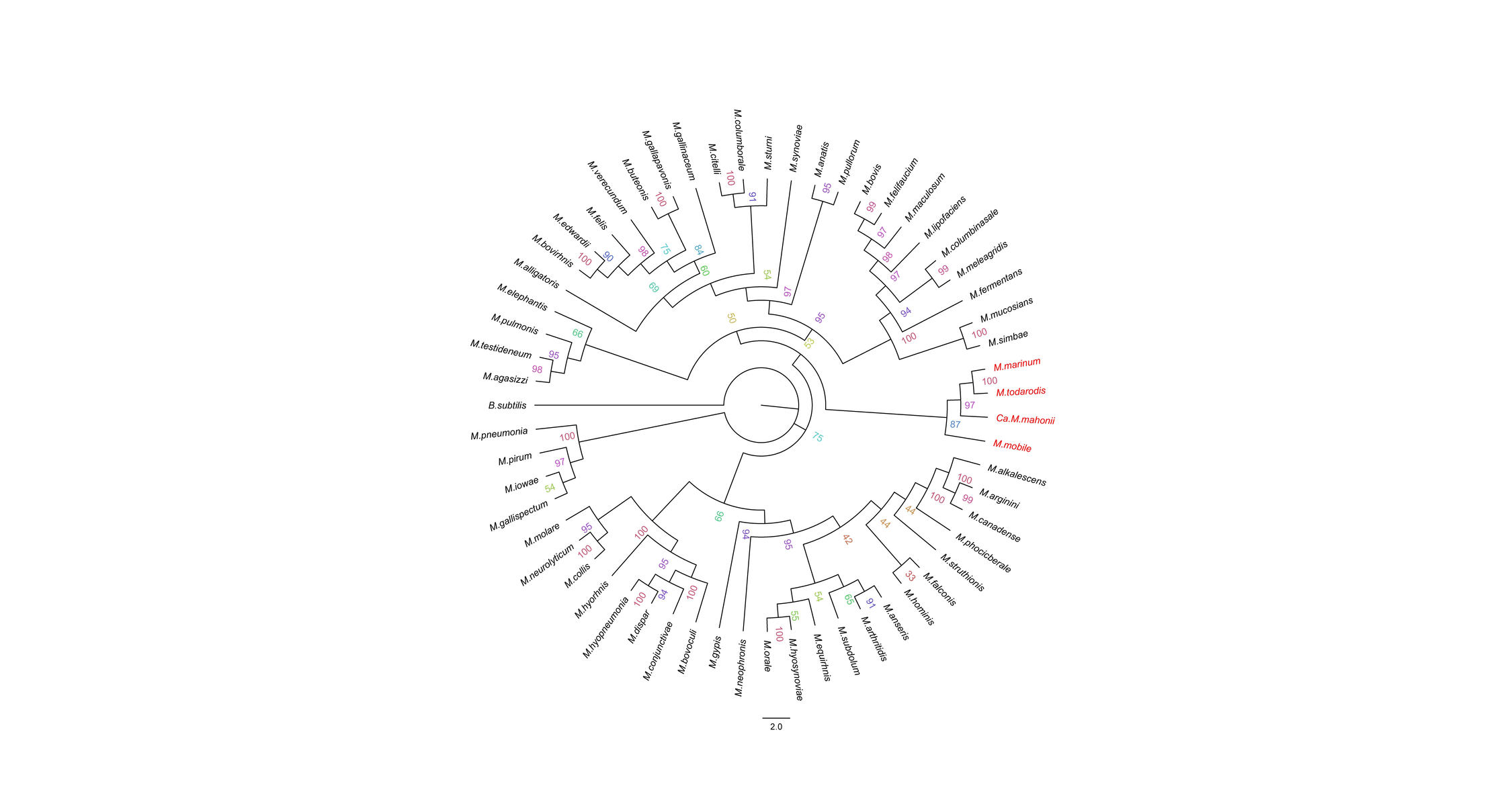

Supplement: S4 Fig — The phylogenetic tree was generated in IQtree with the GTR+F+R5 model, bootstrap percentage values are shown on the tree. (TIF) [file pone.0290305.s004.tif]

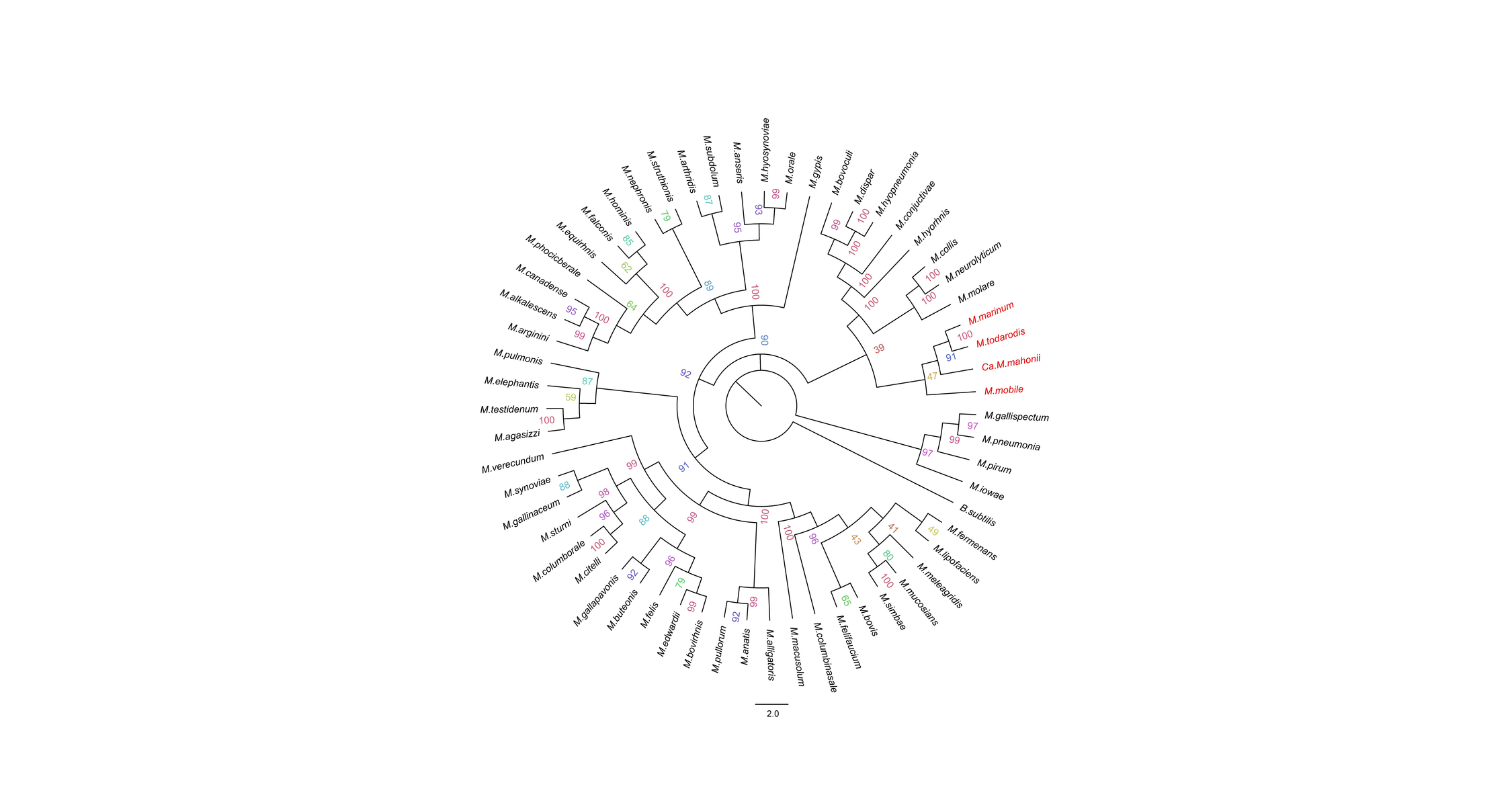

Supplement: S5 Fig — The phylogenetic tree was generated in IQtree with the GTR+F+I+G4 model, bootstrap percentage values are shown on the tree. (TIF) [file pone.0290305.s005.tif]

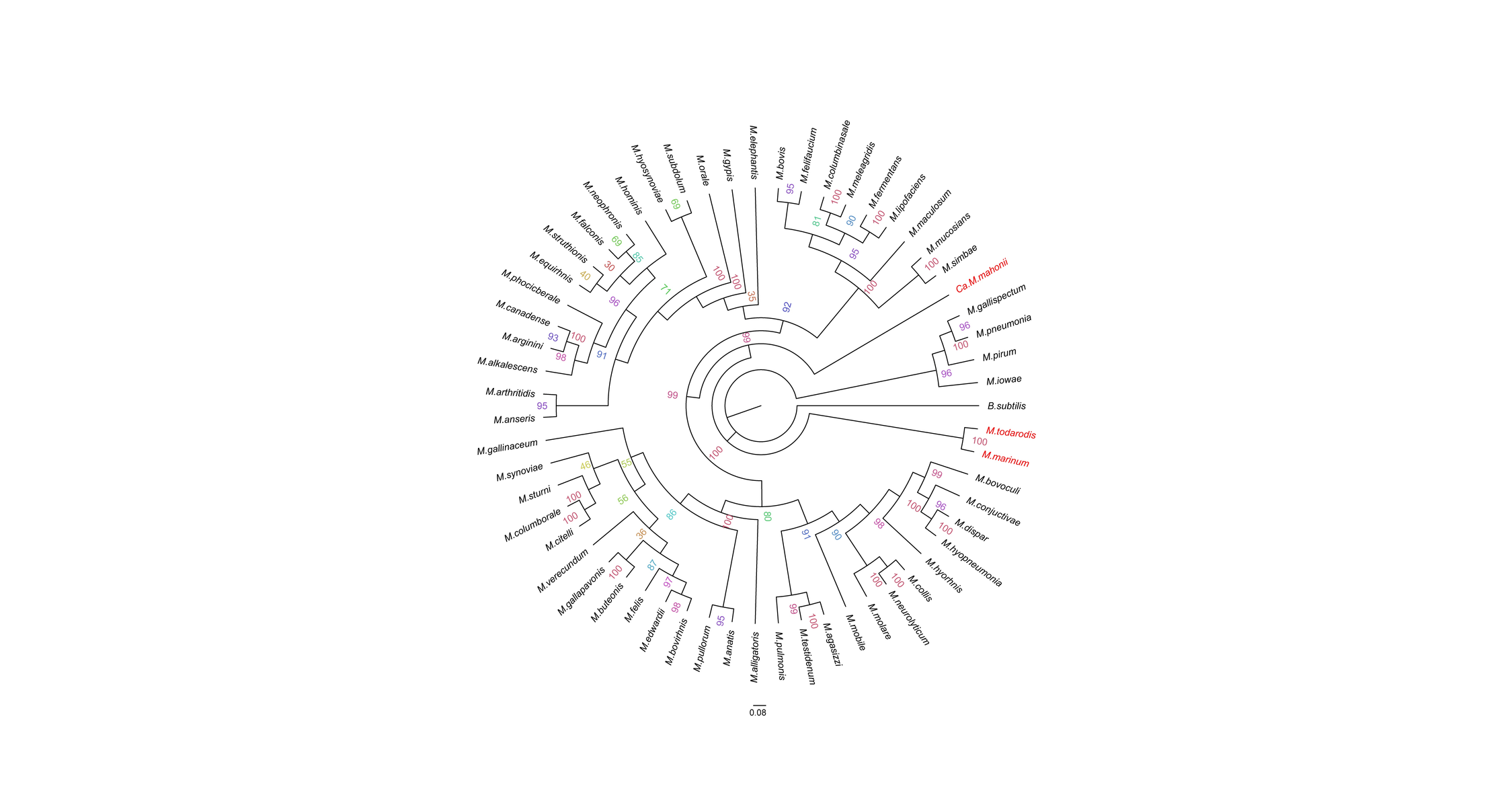

Supplement: S6 Fig — The phylogenetic tree was generated in IQtree with the GTR+F+R5 model, bootstrap percentage values are shown on the tree. (TIF) [file pone.0290305.s006.tif]

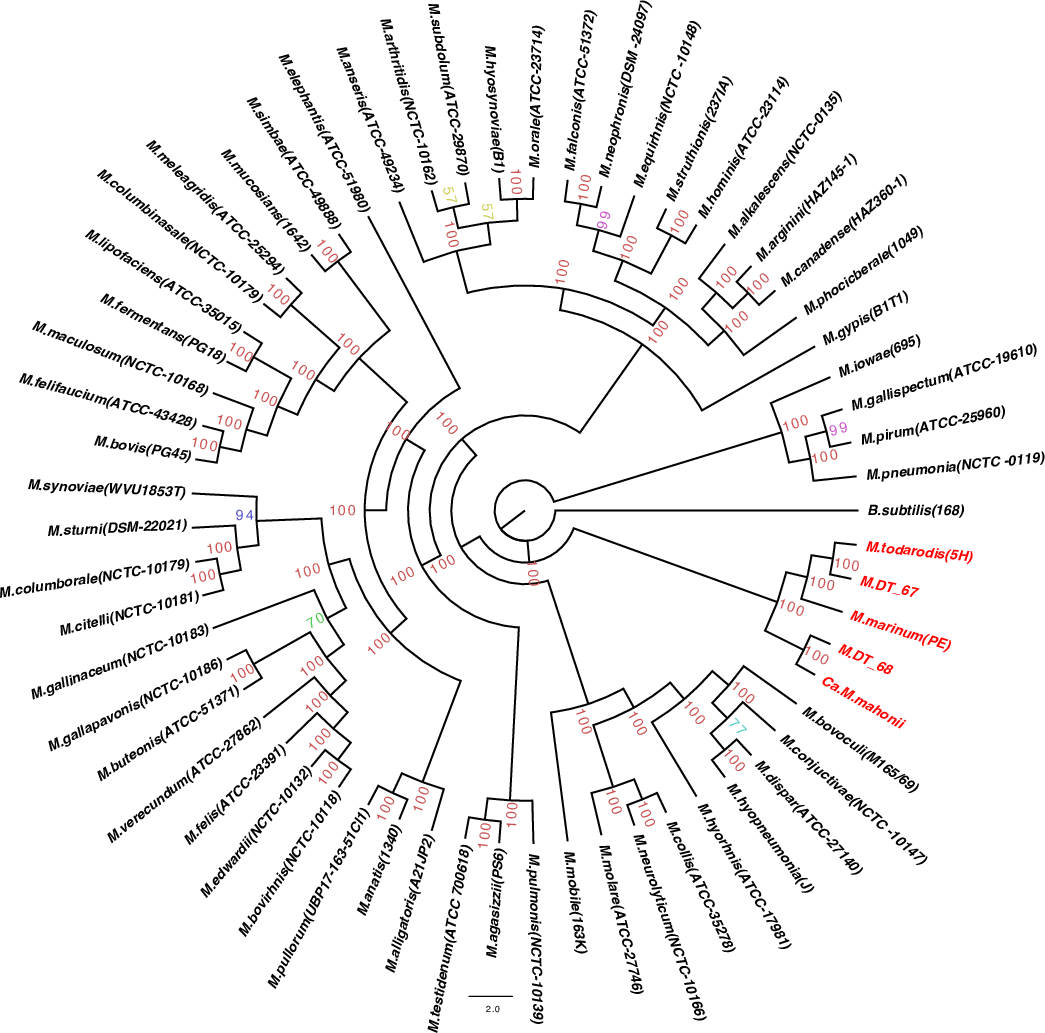

Supplement: S7 Fig — The tree was generated using MrBayes with the GTR+I+G model chosen by JModelTest2. Probability percentage values are shown on the tree. Ca. M. mahonii and other sequences making up the distinct marine clade are shaded red. (TIF) [file pone.0290305.s007.tif]

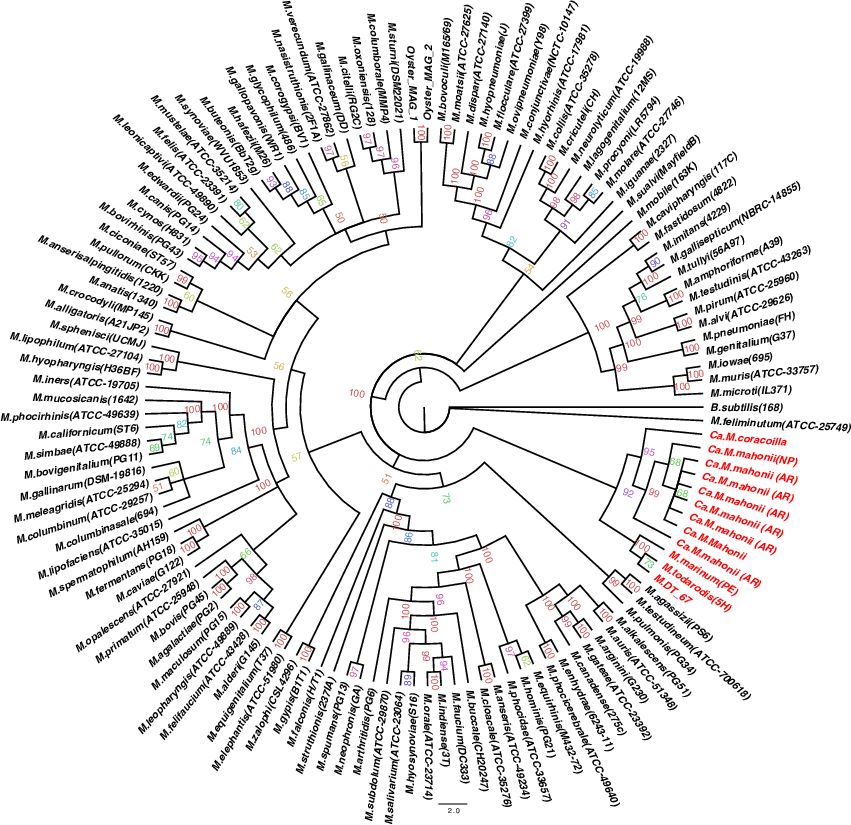

Supplement: S8 Fig — The tree was generated using MrBayes with the GTR+I+G model chosen by JModelTest2. Probability percentage values are shown on the tree. Ca. M. mahonii and other sequences making up the distinct marine clade are shaded red. AR–Argentinian waters samples, NP–North Pacific samples. (TIF) [file pone.0290305.s008.tif]
