# Supplementary material for: Genomic characterization of a novel, widely distributed Mycoplasma species “Candidatus Mycoplasma mahonii” associated with the brittlestar Gorgonocephalus chilensis
Source: PLoS One. 2023 Aug 24;18(8):e0290305. doi: 10.1371/journal.pone.0290305 (PMC10449156; doi:10.1371/journal.pone.0290305)
Supplement: Attachment — Submitted filename: Aroh_response_to_reviewers.docx [file pone.0290305.s021.docx]

Dear Dr. Shastry,

Thank you for the recent decision letter. Comments/responses are given below the comments in blue Calibri font

Reviewer #1: Review of Manuscript: PONE-D-23-03531R1


“Genomic characterization of a novel, widely distributed Mycoplasma Species “Candidatus Mycoplasma mahonii” associated with the brittlestar Gorgonocephalus chilensis

Author(s): Oluchi, Aroh; Halanych, Kenneth; & Liles, Mark

In this reviewed manuscript, the authors have done substantial work to adapt/explain the reviewers’ concerns, particularly those referring to the phylogenetic position of the novel Mycoplasma species and its related taxa. My opinion is that the manuscript has benefited from not-demanded structural changes as well, particularly in the introduction and discussion. I also appreciated a more clear description of the symbiotic relationships between the different Mycoplasma spp. and their hosts. Finally, I have also noticed a higher level of contingency when speaking about the distribution of the species, which I consider positive with the data at hand. Based on the above, I have no further major recommendations for the authors rather than some minor changes which will be most likely corrected in later steps if the manuscript is eventually accepted.

For instance, lines 52- 53: “Pathogenic Mycoplasmas are responsible for numerous respiratory and other infections including pneumonia”. Is not pneumonia a respiratory infection? I would recommend rewording. Or line 60: Salmonid(s)?: Line 123: “search BLAST search tool”; & Line 153: “The second analyses”? To pinpoint few examples. Reviewer #3: Candidatus or Ca. should be in italics. Mycolasma mahoni should not be italicized. Please maintain uniformity. In addition, please check all scientific names for italics

Manuscript has been revised and corrected.

Size and accession number for 16S rRNA genes (Ca. M. mahoni and reference species) used in the study need to be specified.

Information has been added to the supplementary file.

Type species of Mycoplasma need to be shown in the phylogenetic tree
Improvise the figure quality. Resolution of image is poor. Fig. 4 and Fig. 5 are not readable.
Please correct the erroneous genome size given in Fig. 1 (796786 kb).

Figures have been revised accordingly.

Genome of Ca. M. Mahoni is not available in the NCBI database for review.

The genomes are now available under the accessions listed in the manuscript.

Closely related Culturable type strain(s) (if exist) of Mycoplasma under investigation need to be emphasized.

We already mentioned the closely related strains *M. marinum* and *M. todarodis*, however, we have modified the wording to make it clear these are closely related culturable type strains.
